# Isoselective Lactide Ring Opening Polymerisation using [2]Rotaxane Catalysts

**DOI:** 10.1002/anie.201901592

**Published:** 2019-03-28

**Authors:** Jason Y. C. Lim, Nattawut Yuntawattana, Paul D. Beer, Charlotte K. Williams

**Affiliations:** ^1^ Department of Chemistry University of Oxford Chemistry Research Laboratory Mansfield Road Oxford OX1 3TA UK; ^2^ Current address: Institute of Materials Research and Engineering 2 Fusionopolis Way Singapore 138634 Singapore

**Keywords:** isoselectivity, lactide, organocatalysis, ring-opening polymerisation, rotaxane

## Abstract

Polylactide (PLA) is a fully biodegradable and recyclable plastic, produced from a bio‐derived monomer: it is a circular economy plastic. Its properties depend upon its stereochemistry and isotactic PLA shows superior thermal‐mechanical performances. Here, a new means to control tacticity by exploiting rotaxane conformational dynamism is described. Dynamic achiral [2]rotaxanes can show high isoselectivity (P_i_=0.8, 298 K) without requiring any chiral additives and enchain by a chain end control mechanism. The organocatalytic dynamic stereoselectivity is likely applicable to other small‐molecule and polymerization catalyses.

Polylactide (PLA) is a commercially available bio‐derived thermoplastic; its biocompatibility and biodegradability have enabled substitution of petro‐polymers in medical, packaging and fibre applications.^1^ PLA's thermal‐mechanical properties depend upon its microstructure: atactic PLA is amorphous whilst isotactic stereoblock PLA is semi‐crystalline. Stereoblock PLA shows an even higher melting temperature (*T*_m_) than isotactic PLLA.^2^ All tacticities of PLA are produced by the ring opening polymerization (ROP) of lactide. A current challenge is to deliver stereoblock PLA from racemic lactide (*rac*‐LA) using selective catalysis.^3^

Organocatalysts are attractive due to the high polymerization control exhibited and thioureas,^4^ amidines,^5^ phosphazenes^6^ or N‐heterocyclic carbenes (NHCs) also show high rates.^7^ Very few organocatalysts are isoselective (*rac*‐LA) and the most successful are chiral.^8^ For example, binaphthol‐derived phosphoric acid (*k*_D_/*k*_L_ *=*28),^9^ chiral synthetic prolines (*P*_i_=0.87–0.96)^10^ and a chiral thiourea (*P*_i_=0.82)^11^ operate by enantiomorphic site control. Fundamentally this mechanism cannot maintain both high rates and conversions: once the preferred enantiomer is consumed the remaining enantiomer reacts very slowly. The alternative chain end control (CEC) mechanism could overcome this limitation but is under‐developed. It has been successfully applied to various metal complexes^12^ and there are intriguing hints it may operate for a few organocatalysts.6a, 7 Because CEC relies on in operando stereochemical interactions, it is much harder to rationally improve selectivity. We are interested in understanding how catalyst dynamic processes, for example, ligand fluxionality, may enhance stereoselectivity.^12^ We previously reported high isoselectivity yttrium catalysts and correlated ligand fluxionality with stereocontrol.^12^ Okuda and co‐workers showed that fluxional enantiomeric scandium catalysts were heteroselective.^13^ Schaper and co‐workers showed fluxional copper catalysts were isoselective.^14^ So far, related examples of dynamic organocatalysts are unknown.

We reasoned that mechanically interlocked molecules such as rotaxanes could allow for dynamic stereocontrol as macrocycle and axle components can be stimulated to move independently. Such macrocycle/axle dynamic behavior underpins many of their applications, for example, in molecular recognition/sensing,^15^ molecular machines,^16^ switchable catalysis^17^ and artificial ribosome mimics.^18^ To test their potential for *rac*‐LA ROP, rotaxanes **1**, **2** and **3** were targeted (Figure 1, SI for synthesis). Each comprises a crown ether macrocycle and an axle, the latter containing both an ammonium and a thiourea or triazole group. In this form, the macrocycle resides over the protonated ammonium group secured by strong intramolecular hydrogen bonding (Figure S3, S10, S14).^19^ After deprotonation, the amine interacts weakly with the macrocycle allowing its free movement along the axle. This protonation‐dependent dynamic behavior also highlights their potential to be switched “on” for catalysis.

**Figure 1:**
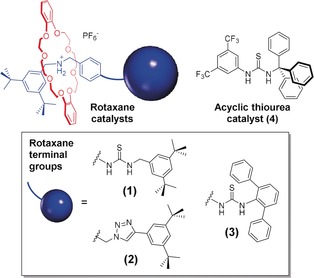
Structures of [2]rotaxane catalysts and non‐interlocked control compounds.

Rotaxanes **1**–**3** were all inactive for *rac*‐LA ROP, even with added benzyl alcohol, consistent with previous reports that thioureas alone cannot activate lactide to attack by alcohols.4a Neutral rotaxanes, deprotonated in situ by addition of base, were active in presence of benzyl alcohol (Table 1, entries 1, 7 and 8). The rates differ, with **1** reaching 80 % after 4 d, whilst **2** and **3** achieved high conversions within 12 h. All catalysts showed high polymerization control and produced PLA with predictable molar masses (*M*_n_), linear evolution of *M*_n_ with conversion, and narrow dispersity (*Ð*<1.20) (Figure 2 A, S31–S34). End‐group analysis, using ESI mass spectrometry, showed a single set of peaks corresponding to benzyl ester end‐capped chains (Figure S28–S30). Transesterification side reactions were not observed for **1** and **3**, as evidenced by the 144 peak separations, whilst peak separations of 72 for **2**, indicates transesterification. It is noted that neutral rotaxane **2** is the first example of a triazole‐containing organocatalyst for lactide ROP.

**Figure 2:**
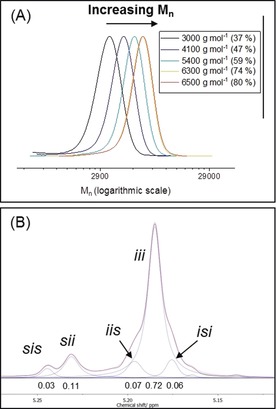
A) Evolution of molar mass with conversion for polymerizations catalyzed by **1**. B) ^1^H{^1^H} NMR spectrum with tetrads labelled. Reaction conditions: [LA]/[**1**]/[KN(SiMe_3_)_2_]/[BnOH]=50:1:1:1, [LA]=1.0 m, THF, 298 K (*P*_i_=0.81).

**Table 1:** Polymerisation of *rac*‐lactide with rotaxane catalysts.

Entry^[a]^	Catalyst	[LA]/[cat.]/[base]/[BnOH]	*t* [h]	Conv.^[b]^ [%]	*M*_n_^[c]^ [kg mol^−1^]	*M*_n_^calc.^ [kg mol^−1^]	*Ð* ^[c]^	*P*_i_^[d]^
1	**1**	50:1:1:1	96	80	6.5	5.8	1.08	0.81±0.03
2	–	50:0:1:1	1	99	8.3	7.1	1.72	0.56±0.10
3	**1**	50:1:0:1	48	0	–	–	–	–
4	**1**	50:1:1:0	48	0	–	–	–	–
5	**1**	100:1:2:2	1	99	8.8	7.1	1.47	0.54±0.06
6	**1**	50:1:1:1 (323 K)	72	80	5.4	5.8	1.11	0.66±0.02
7	**2**	50:1:1:1	11	80	5.8	5.8	1.16	0.66±0.03
8	**3**	50:1:1:1	3	90	6.2	6.5	1.13	0.73±0.02
9	**4**	50:1:1:1	5	98	8.1	7.1	1.16	0.69±0.01

[a] Polymerization conditions: [LA]=1.0 m, THF, 298 K, unless stated. [b] Determined by integrating ^1^H NMR spectrum (LA, 4.96–5.04 ppm; PLA, 5.10–5.22 ppm). [c] Determined by SEC analysis, in THF, and applying a correction factor of 0.58 to values.^24^ [d] Determined from the ^1^H{^1^H} NMR by integration of normalized methine tetrads and Bernoullian statistics (Figure S35–S41, SI).^25^

Three components are required for control: rotaxane, base and alcohol. Polymerizations conducted without rotaxane were rapid but uncontrolled yielding atactic PLA (Table 1, entry 2). Reactions without alcohol were inactive (Table 1, entry 4). Under the appropriate conditions, **1** produced highly isotactic PLA (*P*_i_=0.81; Figure 2 B, S35). Replacing the thiourea group of **1** with the weaker hydrogen bond‐donating triazole in **2** significantly reduces stereocontrol (*P*_i_=0.66; Figure S39). Thiourea accessibility also appears to be important, as **3** shows lower stereocontrol (*P*_i_=0.73, Figure S40). In all cases, analysis of defect tetrad resonances, in the methine region of the ^1^H{^1^H} NMR spectra, suggests a likely dominant CEC mechanism (see Figure S35, SI).^20^, ^21^ Despite a small extent of epimerization occurring during the reaction (Figure S27), the highest stereocontrol, achieved by **1**, matches values for chiral organocatalysts,^8^ urea/alkoxide systems^22^ or organometallic catalysts.^23^ Perhaps more interesting is the finding that the rotaxane structures, and dynamism, are inherent to stereocontrol. Under identical conditions, the macrocycle alone yields only atactic PLA, while the protonated thiourea axle is inactive (SI). The possibility that isoselectivity results from only thiourea steric congestion can also be ruled out because the equivalent acyclic catalyst **4** shows lower selectivity (*P*_i_=0.69, Figure S41). Furthermore, increased thiourea steric hindrance, in **3**, reduces stereoselectivity. These observations point towards an alternative stereocontrol.

The NMR spectra of neutral rotaxanes were compared to understand catalyst structure in more detail. Addition of equimolar base to **1**, results in upfield shifts to resonances H_3_ and H_4_, which are immediately adjacent to the ammonium group (Figure 3). Such shifts indicate formation of a neutral amine group. At the same time, thiourea resonances H_α_ and H_β_ shift downfield, consistent with enhanced thiourea‐macrocycle interactions. No significant changes were observed after the addition of benzyl alcohol (Figure S44). Significant broadening of all other resonances indicates the macrocycle is moving along the axle slower than the NMR timescale. Adding base to **2**, also deprotonates the ammonium group (Figure S45). The remaining signals are sharp suggesting the rate of macrocycle movement along the axle is faster than for **1**. The ROESY NMR spectrum of (neutral) **2** provides unequivocal evidence of macrocycle movement between amine and triazole sites, evident from the numerous cross‐peaks between the macrocycle protons and those arising from the aromatic stopper units on both ends of the axle (Figure S49).

**Figure 3:**
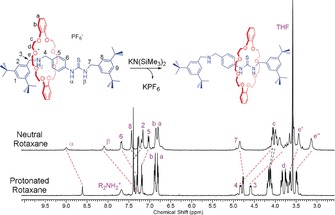
Stacked ^1^H NMR spectra of protonated and neutral rotaxane **1** ([**1**]=2 mm, *T*=298 K, *d*_8_THF).

Intense ROESY signals correlate with the macrocycle being preferentially located at the amine site, likely due to its greater hydrogen bonding acidity compared to the triazole. In contrast, neutral **1** would be expected to show favorable interactions with both thiourea and amine sites. Its ROESY NMR reveals stronger through‐space interactions between the macrocycle and the thiourea (Figure S47). Accordingly, stronger macrocycle‐thiourea hydrogen‐bonding may slow macrocycle translation along the axle and broaden the NMR spectrum. The ^1^H NMR spectrum for neutral **3** also indicates the axle amine group was deprotonated (Figure S45). ROESY NMR could not confirm the macrocycle site occupancy due to the complex, overlapping aromatic resonances (Figure S50). Nonetheless, the bulky stopper group may be expected to hinder thiourea–macrocycle interactions and favour macrocycle occupation of the amine site.

Control experiments support the neutral rotaxane being the true catalyst (Scheme 1 A). Neither potassium hexafluorophosphate, the amine by‐product (N(SiMe_3_)_2_H) nor benzyl alcohol initiate polymerization, either individually or in combinations (Section S3.2, SI). The free axles of **1** and **2** were also inactive. A strong base is necessary for rotaxane deprotonation: replacing KN(SiMe_3_)_2_ with DABCO (a weaker base) results only in limited ROP (7 % after 120 hours). To rule out formation of rotaxane‐potassium complexes, the ^1^H NMR spectra of the potassium and sodium salts of **1** were compared. Both spectra showed identical chemical shifts for all resonances (Figure S51). As K^+^/Na^+^ coordination would be expected to give different chemical shifts, the active species are unlikely to be Group 1 complexes. Overall, the data indicate that the catalyst is a neutral rotaxane and DOSY NMR indicates it is monomeric under the conditions of catalysis (Figure S48).

**Scheme 1:**
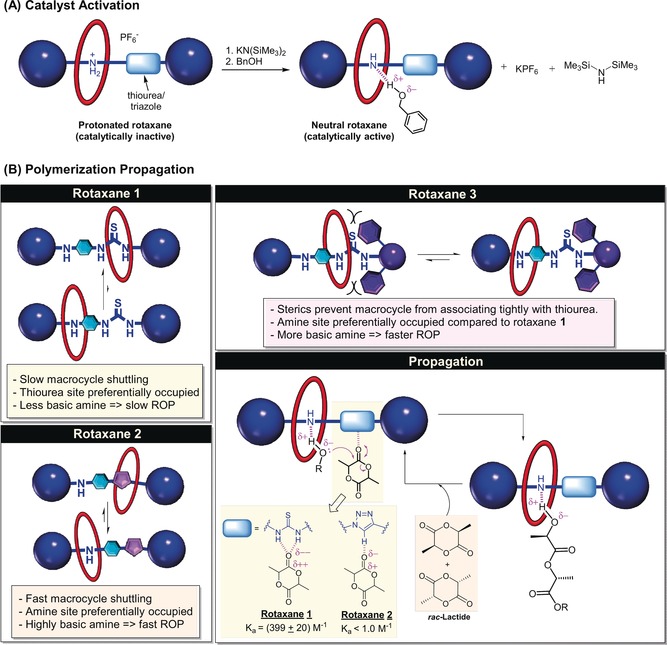
Proposed mechanisms of A) [2]rotaxane catalyst activation and B) lactide ROP with **1**, **2** and **3**.

To rationalize the differing catalytic performances, a polymerization pathway is proposed inspired by the two‐component thiourea/amine organocatalysis literature (Scheme 1 B).4a,4b Neutral **1**/**2** feature thiourea/triazole groups which activate the lactide monomer and amine groups to activate benzyl alcohol. This mode of initiator/ monomer activation is only possible if the macrocycle is free to move along the axle. Polymerization is initiated, and propagated, by an activated monomer mechanism. Importantly, for these rotaxanes, the basicity of the axle secondary amine is considerably enhanced by interaction with the crown ether macrocycle component compared to the free neutral axle, as the stability of the crown ether‐R_2_NH_2_^+^ conjugate acid upon protonation strongly favours its formation thermodynamically.19c, 26 Accordingly, the activated benzyl alcohol attacks the coordinated lactide forming an α‐hydroxyl‐ω‐ester which is subsequently (re)activated and attacks another lactide. In line with this, the relative rates correlate with rotaxane amine basicity, and hence capacity for alcohol activation. Because the rotaxanes also feature other macrocycle coordination sites, when these sites are strongly coordinating the amine basicity is reduced.^27^ For **2** and **3**, macrocycles occupy the amine site, increasing its basicity and accelerating rates (Scheme 1 B).

Only **1** shows high isoselectivity and operates by an unusual chain end control mechanism. To investigate further, the rotaxane‐lactide binding constants were determined by ^1^H NMR titrations. **1** shows a higher binding constant (*K*_a_≈400 m ^−1^) than **2** (*K*_a_<1 m ^−1^) (Section S4.2, SI).^28^ From the aforementioned ROESY studies, it also shows significantly slower macrocycle shuttling. It is tentatively proposed that both the strong binding and slow shuttling allow for the preferential orientation of the growing polymer chain and lactide resulting in the isoselectivity.

In conclusion, these are the first rotaxane catalysts for lactide ROP and they show isoselectivity. The polymerization selectivity correlates with macrocycle–axle translocation rates and with sterically‐accessible, strong monomer coordination sites. The conformational dynamism appears to be responsible for both rates and stereoselectivity and provides a new means by which to control tacticity. This new type of stereocontrol is expected to be applicable to transesterifications, transamidations and other ring‐opening polymerizations of epoxides, cyclic carbonates and lactones.

## Conflict of interest

The authors declare no conflict of interest.

## Supporting information

As a service to our authors and readers, this journal provides supporting information supplied by the authors. Such materials are peer reviewed and may be re‐organized for online delivery, but are not copy‐edited or typeset. Technical support issues arising from supporting information (other than missing files) should be addressed to the authors.

Supplementary
