# Supplementary material for: Isoselective Lactide Ring Opening Polymerisation using [2]Rotaxane Catalysts
Source: Angew Chem Int Ed Engl. 2019 Mar 28;58(18):6007–11. doi: 10.1002/anie.201901592 (PMC6519244; doi:10.1002/anie.201901592)
Supplement: Supplementary file 1 — Supplementary [file ANIE-58-6007-s001.pdf]

Angewandte Chemie
Eine Zeitschrift der Gesellschaft Deutscher Chemiker

Supporting Information

Isoselective Lactide Ring Opening Polymerisation using [2]Rotaxane Catalysts

Jason Y. C. Lim, Nattawut Yuntawattana, Paul D. Beer, and Charlotte K. Williams**

anie_201901592_sm_miscellaneous_information.pdf

<u>Contents</u>

S1 General Procedures **S2**

S2 Experimental **S3**

S2.1 Synthesis of Rotaxane **1** S3

S2.2 Synthesis of Rotaxane **2** S6

S2.3 Synthesis of Rotaxane **3** S9

S2.4 Synthesis of Catalyst **4** S10

S2.5 Synthesis of Thiourea-containing Free Axle S11

S2.6 Synthesis of Triazole-containing Free Axle S12

S2.7 Typical polymerization procedure with rotaxanes **1-3** S12

S2.8 Spectral Characterization of Catalysts S14

S3 Additional Data for Polymerization Experiments **S27**

S3.1 Spectra and characterization of PLA S27

S3.2 Additional Polymerization Experiments S35

S4 Additional NMR Data **S37**

S4.1 Additional Spectra S37

S4.2 ^{1}H NMR Titration Experiments S41

S5 References **S42**

S1. General Procedures

General procedure

All chemical reagents were commercial available and purchased from commercial sources (Aldrich, Fisher and Fluorochem) and used as received unless stated otherwise. All solvents used in the reactions were collected from solvent purification system (SPS) machine and degassed three times using freeze-pump thaw process before use. Tetrahydrofuran was distilled over Na/benzophenone under a nitrogen atmosphere and degassed three times before use. d_8-THF was degassed three times and stored over activated molecular sieves under nitrogen. The *rac*-lactide monomer was recrystallized from a hot anhydrous toluene and sublimed three times before use.

Chromatography: Thin layer chromatography was performed using Merck aluminium-backed DC 60 F_{254} 0.2 mm silica precoated plates, which were visualised with UV fluorescence and staining with potassium(VII) manganite. Silica gel column chromatography was undertaken using silica gel (particle size: 40-63 μm); preparative TLC plates were performed using Analtech glass-backed pre-coated plates (20 × 20 cm, 0.1 cm silica thickness); size-exclusion chromatography was performed using Bio-rad S-X3 styrene divinylbenzene beads with 3 % cross-linkage and 40-80 μM bead size.

NMR spectroscopy: NMR spectra were recorded on Bruker AVIII HD Nanobay 400 MHz, Bruker AVIII 500 MHz and Bruker AVIII 500 MHz (with 13C cryoprobe) NMR spectrometers. The following abbreviations are used in the report of spectra: s, singlet; d, doublet, dd: doublet of doublets; t, triplet; q, quartet; quin; quintet; sex, sextet; sep, septet; m, multiplet.

Gel permeation chromatography: The molecular weights and dispersities were recorded on an Agilent PL GPC-50 instrument, with HPLC grade THF, at 30°C and a flow rate of 1.0 mL/min. In all cases, near monodisperse polystyrene standards were used for calibration. The samples were prepared by dissolving ca. 20 mg of polymer in THF, and filtering through a 2 μm PTFE filter before injection. The molecular weight of the resultant PLA was corrected by the Mark-Houwink factor of 0.58.[1]

High-resolution mass spectrometry: HRMS were performed on an Agilent 7200 quadrupole time of flight (Q-ToF) instrument equipped with a direct insertion probe supplied by Scientific instrument Manufacturer (SIM) GmbH using electron ionization (EI) as an ionization technique. Instrument control and data processing were performed using Agilent MassHunter software.

S2. Experimental

S2.1 Synthesis of Rotaxane 1

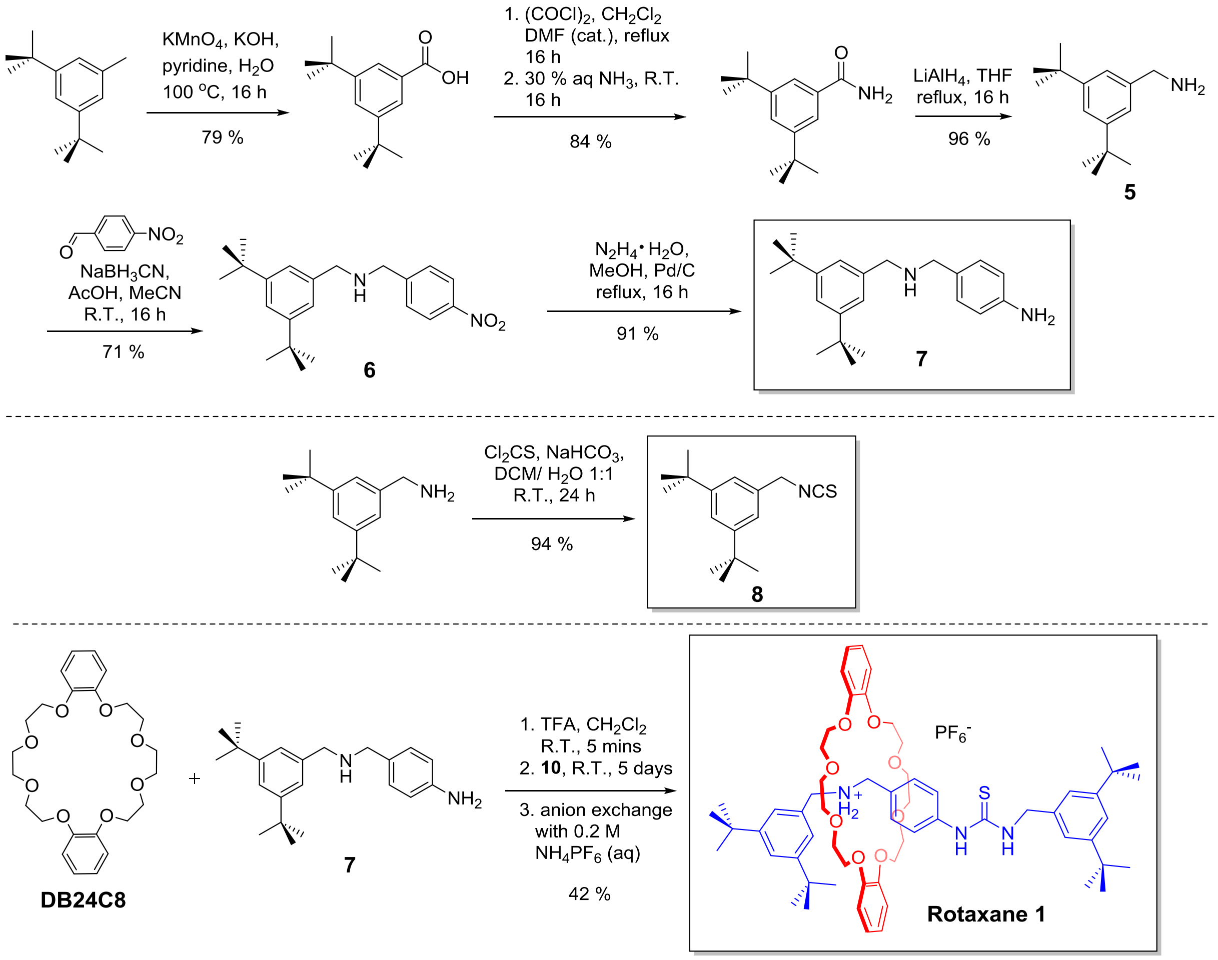

Scheme 1. Synthesis of rotaxane **1**.

3,5-tert-butylbenzoic acid was synthesized as reported[2] and 3,5-tert-butylbenzamide and 3,5-tert-butyl-benzylamine **5** were following a reported procedure.[3]

The following procedures document those for novel compounds.

<u>Nitrobenzene Intermediate **6**</u>

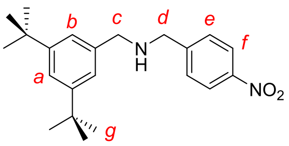

3,5-tert-butyl-benzylamine **7** (500 mg, 2.28 mmol) and 4-nitrobenzaldehyde (313 mg, 2.08 mmol) were dissolved in dry acetonitrile (10 mL). A drop of glacial acetic acid was added and the reaction was stirred at room temperature for 2 hours. Sodium cyanoborohydride (392 mg, 6.24 mmol) was then added portionwise, followed by glacial acetic acid (0.24 mL, 4.2 mmol) and the reaction was stirred overnight. Thereafter, 1 M NaOH (aq.) (10 mL) was added to the reaction and stirred vigorously for 15 minutes to give a solution, before diethyl ether (20 mL) was added. The aqueous

and organic phases were separated and the aqueous layer extracted with diethyl ether (2 x 20 mL). The combined organics were washed with brine (20 mL) and dried with anhydrous $MgSO_4$. The crude reaction mixture was purified by silica gel column chromatography (eluent: ethyl acetate/ dichloromethane 1: 9 *v/v*) to afford the target compound as a viscous yellow oil (473 mg, 71 %).

^{1}H NMR (400 MHz, $CDCl_3$): δ 8.21 (2H, d, $^3J_{HH}$ = 7.0 Hz, H_f), 7.55 (2H, d, $^3J_{HH}$ = 7.0 Hz, H_e), 7.36 (1H, t, $^4J_{HH}$ = 1.2 Hz, H_a), 7.17 (2H, d, $^4J_{HH}$ = 1.2 Hz, H_b), 3.96 (2H, s, H_d), 3.82 (2H, s, H_c), 1.34 (18H, s, H_g).

^{13}C NMR (100 MHz, $CDCl_3$): δ 151.0 (Ar***C***), 147.9 (Ar***C***-NO_2), 147.1 (Ar***C***), 138.6 (Ar***C***), 128.8 (Ar***C***H), 123.6 (Ar***C***H), 122.3 (Ar***C***H), 121.4 (Ar***C***H), 53.8 (NH***C***H_2), 52.3 (***C***H_2NH), 34.8 (***C***CH_3), 31.5 (C***C***H_3)

HRMS (ESI) Calc. ($C_{22}H_{31}N_2O_2$) m/z = 355.2380 $[M + H]^+$; Found m/z = 355.2380.

<u>Axle aniline precursor **7**</u>

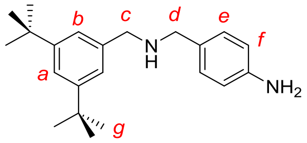

Warning: Hydrazine monohydrate used in this reaction is highly toxic and explosive when dry. Care must be taken to perform the reaction in a well-ventilated fume cupboard and never heat the reaction to dryness.

Nitrobenzene precursor **6** (300 mg, 0.85 mmol) was dissolved in methanol (15 mL) and palladium on carbon (10 % weight, 30 mg) was added to the reaction, slowly, under a stream of nitrogen (*Warning: beware of sparks upon addition of Pd/C to methanol, always perform under* N_2). Thereafter, hydrazine monohydrate (0.82 mL, 17.0 mmol) was added, portionwise, to the vigorously-stirred reaction, which was then heated, under reflux, overnight. Upon cooling to room temperature, the black suspension was filtered over a plug of celite and the product was eluted with methanol (c.a. 100 mL). The solvent was removed *in vacuo* and the residue dissolved in dichloromethane (30 mL) and washed with saturated aqueous sodium bicarbonate (20 mL) and brine (20 mL) then dried with anhydrous $MgSO_4$. Removal of solvent *in vacuo* afforded the target compound as a colourless viscous oil (251 mg, 91 %) in good purity. The product was used directly in the next step without further purification.

^{1}H NMR (400 MHz, $CDCl_3$): δ 7.36 (1H, t, $^4J_{HH}$ = 1.1 Hz, H_a), 7.25 (2H, d, $^4J_{HH}$ = 1.1 Hz, H_b), 7.19 (2H, d, $^3J_{HH}$ = 6.6 Hz, H_e), 6.65 (2H, d, $^3J_{HH}$ = 6.6 Hz, H_f), 3.84 (2H, s, H_c), 3.75 (2H, s, H_d), 3.66 (2H, br. s, NH_2), 1.33 (18H, s, H_g).

^{13}C NMR (100 MHz, $CDCl_3$): δ 151.2 (Ar***C***), 146.3 (Ar***C***-NH_2), 130.6 (Ar***C***H), 123.5 (Ar***C***H), 122.0 (Ar***C***H), 115.1 (Ar***C***H), 51.2 (***C***H_2NH), 50.4 (NH***C***H_2), 34.9 (***C***CH_3), 31.5 (C***C***H_3) (2 Ar***C*** peaks not seen due to overlap)

HRMS (ESI) Calc. ($C_{22}H_{33}N_2$) m/z = 325.2638 $[M + H]^+$; Found m/z = 325.2640.

3,5-tert-butyl-benzylisothiocyanate **8**

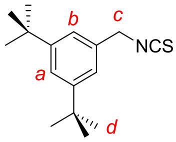

Warning: Thiophosgene, used in this reaction, is a highly-toxic and malodorous red liquid, and must always be handled in a well-ventilated fume cupboard. All excess reagents and glassware/ apparatus in contact with the compound should be thoroughly quenched with excess cold dilute aqueous ammonia before disposal.

Benzylamine **5** (390 mg, 1.78 mmol) was dissolved in dichloromethane/ water 1:1 *v/v* (6 mL). To the vigorously-stirred biphasic mixture was added sodium bicarbonate (750 mg, 8.93 mmol), and the reaction chilled to 0 ºC in an ice bath before thiophosgene (0.14 mL, 1.78 mmol) was added dropwise over 5 minutes. The reaction was warmed up to ambient temperature before stirring overnight. Dichloromethane (30 mL) and water (10 mL) was added to the reaction and the organic layer separated. The organic layer was washed successively with saturated aqueous sodium bicarbonate (20 mL), water (20 mL) and brine (20 mL) and dried with $MgSO_4$. Purification by passing the orange crude product through a short silica plug (eluent: dichloromethane) afforded the product as a pale yellow oil (436 mg, 94 %) which solidified upon standing for a few days.

^{1}H NMR (400 MHz, $CDCl_3$): δ 7.41 (1H, t, $^4J_{HH}$ = 1.2 Hz, H_a), 7.15 (2H, d, $^4J_{HH}$ = 1.2 Hz, H_b), 4.71 (2H, s, H_c), 1.35 (18H, s, H_d).

^{13}C NMR (100 MHz, $CDCl_3$): δ 151.7 (Ar***C***), 133.4 (Ar***C***), 132.1 (N***C***S), 122.4 (Ar***C***H), 121.1 (Ar***C***H), 49.2 (***C***H_2NCS), 34.9 (***C***CH_3), 31.4 (C***C***H_3).

HRMS (EI) Calc. ($C_{16}H_{23}NS$) m/z = 261.1538 $[M]^+$; Found m/z = 261.1546.

Rotaxane **1**

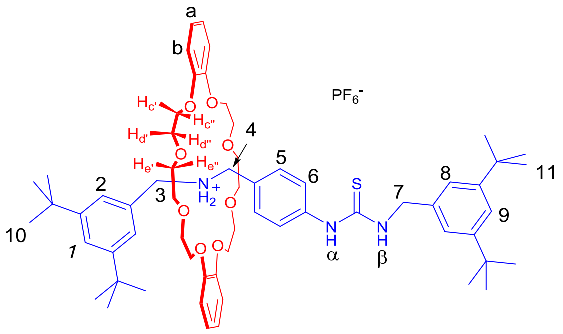

Aniline axle precursor **7** (150 mg, 0.46 mmol) and commercially-available dibenzo[24]crown[8] (DB24C8) macrocycle (415 mg, 0.92 mmol) were dissolved in anhydrous dichloromethane (1.0 mL) under vigorous sonication before neat trifluoroacetic acid (35 μL, 0.46 mmol) was added. After stirring for 15 minutes, isothiocyanate **10** (180 mg, 0.69 mmol) was added portionwise and the reaction stirred at room temperature for 5 days. The reaction was diluted with dichloromethane (30 mL) and washed successively with saturated aqueous sodium bicarbonate (20 mL) and water (20 mL) then dried with $MgSO_4$. The beige crude solid, obtained after rotary evaporation of the solvent, was purified by silica preparatory thin layer chromatography (eluent: 4 % methanol in dichloromethane *v/v*). After extracting the purified rotaxane from the silica gel using 10 % methanol in dichloromethane *v/v* after stirring overnight, the rotaxane was further purified by size-exclusion chromatography (eluent: chloroform).

Anion exchange was performed by dissolving the purified rotaxane in chloroform (30 mL) and washing successively with 0.2 M NH_4PF_6 (aq.) (8 x 15 mL) then water (20 mL). The organic layer was dried with $MgSO_4$ and solvent was removed to afford the target rotaxane as a pale beige solid (229 mg, 42 %). The rotaxane was dried thoroughly *in vacuo* and in a vacuum dessicator over P_2O_5 for at least 3 days, and stored in a N_2-filled glovebox in between usage.

^{1}H NMR (500 MHz, d_6-acetone): 8.73 (1H, br. s, thiourea NH_α), 7.73 (2H, br. s, NH_2^+), 7.48 (1H, t, $^4J_{HH}$ = 1.8 Hz, H_1), 7.45 (2H, d, $^4J_{HH}$ = 1.8 Hz, H_2), 7.42 (1H, t, $^4J_{HH}$ = 1.8 Hz, H_9), 7.31 (2H, d, $^4J_{HH}$ = 1.8 Hz, H_8), 7.26 (2H, d, $^3J_{HH}$ = 6.8 Hz, H_5), 7.19-7.22 (2H, m, H_6), 6.90-6.92 (4H, m, H_b), 6.83-6.85 (4H, m, H_a), 4.87-4.89 (2H, m, H_3), 4.83 (2H, d, $^3J_{HH}$ = 3.9 Hz, H_7), 4.70-4.72 (2H, m, H_4), 4.16-4.23 (8H, m, H_c), 3.90-3.94 (4H, m, $H_{d''}$), 3.80-3.84 (4H, m, $H_{d'}$), 3.71-3.75 (4H, m, $H_{e''}$), 3.57-3.60 (4H, m, $H_{e'}$), 1.33 (18H, s, H_{10}), 1.23 (18H, s, H_{11}).

^{13}C NMR (126 MHz, d_6-acetone): 181.3, 151.2, 150.8, 147.6, 139.9, 137.7, 131.8, 130.1, 127.7, 123.8, 123.3, 122.8, 122.2, 121.3, 121.1, 112.5, 70.5, 70.2, 68.0, 52.8, 52.1, 48.6, 34.6, 34.5, 30.9, 30.8.

^{19}F NMR (470 MHz, d_6-acetone): -72.3 (d, $^1J_{FP}$ = 706 Hz).

^{31}P NMR (202 MHz, d_6-acetone): -144.3 (sept., $^1J_{PF}$ = 706 Hz).

HRMS (ESI): Calc. ($C_{62}H_{88}N_3O_8\,^{32}S$) m/z = 1034.6292 $[M]^+$; Found m/z = 1034.6293.

S2.2 Synthesis of Rotaxane 2

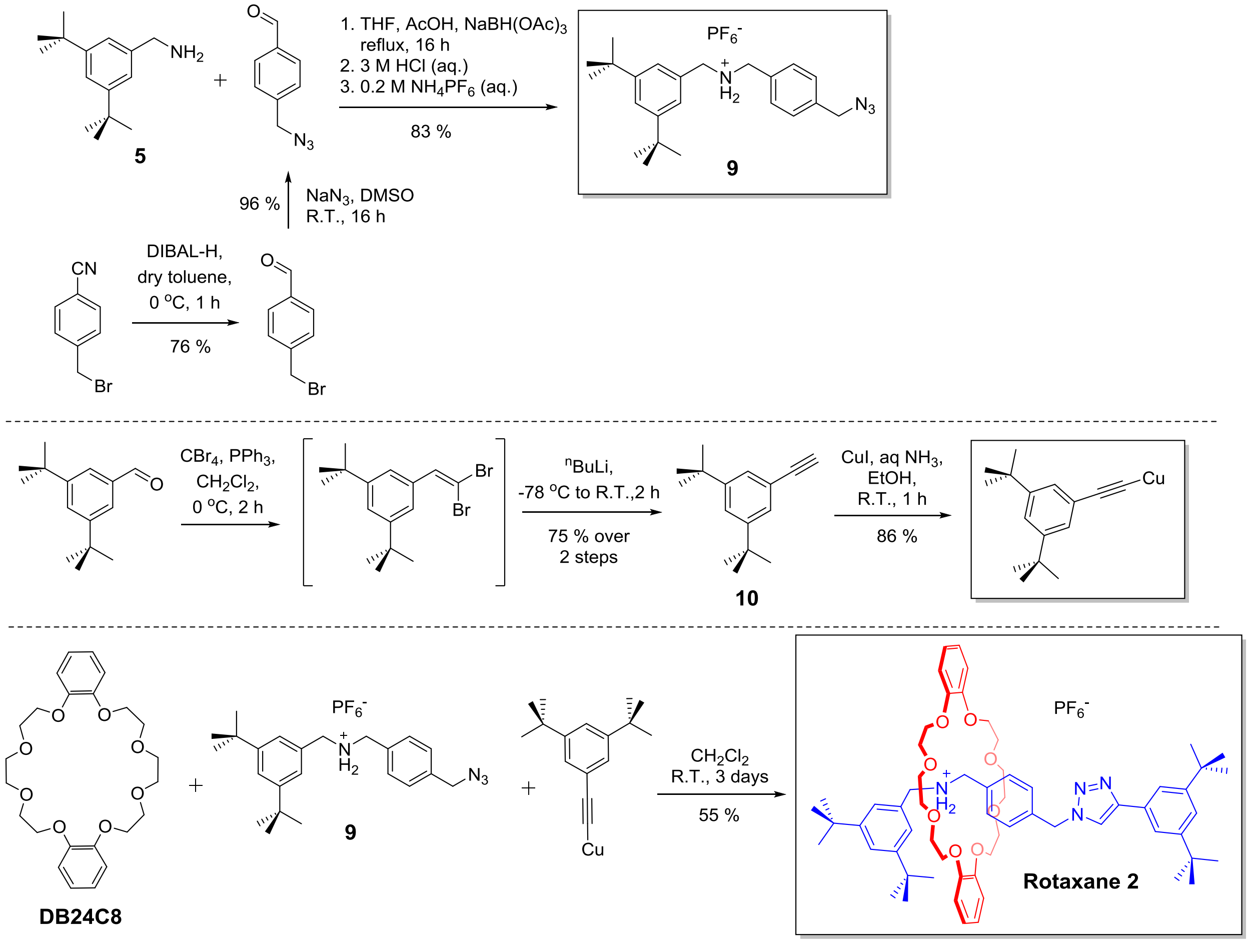

Scheme 2. Synthesis of rotaxane **2**.

4-(Azidomethyl)benzaldehyde was synthesized from 4-(bromomethyl)benzonitrile following a reported procedure,[4] and 3,5-tert-butylbenzaldehyde was synthesized from 3,5-tert-butylbenzoic acid by reduction using $LiAlH_4$ to form the primary alcohol,[5] followed by oxidation using pyridinium chlorochromate.

Axle azide precursor **9**

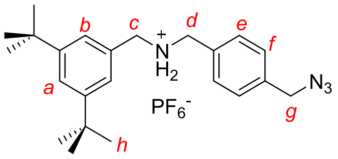

Benzylamine **5** (480 mg, 2.18 mmol) and 4-(azidomethyl)benzaldehyde (335 mg, 2.08 mmol) were dissolved in dry THF (15 mL). Glacial acetic acid (0.12 mL, 2.08 mmol) was added, dropwise, to the pale yellow solution whereupon a white suspension formed. After stirring for 15 minutes, sodium tris(acetoxy)borohydride (882 mg, 4.16 mmol) was added, portionwise, and the reaction was stirred at room temperature for 1 hour, before being heated at reflux overnight. Upon cooling to room temperature, the crude reaction mixture was concentrated in a rotary evaporator until c.a. 5 mL volume and was then poured into 3 M HCl (aq.) (150 mL) to give a white precipitate. The suspension was left to stand for 15 minutes, then filtered under vacuum, and the residue washed with water (c.a. 50 mL). Anion exchange to the PF_6^- salt was achieved by dissolving the resulting white solid in a minimum quantity of acetone and pouring into 0.25 M NH_4PF_6 (aq.) (100 mL) to afford a sticky solid. The cloudy aqueous layer was extracted with ethyl acetate (3 x 25 mL) and the organic layer was dried with $MgSO_4$. Solvent removal *in vacuo* afforded the product as a beige solid (885 mg, 83 %).

^{1}H NMR (500 MHz, *d*$_6$-acetone): 8.70 (2H, br. s, NH_2^+), 7.62 (2H, d, $^3J_{HH}$ = 8.0 Hz, H_e), 7.57 (1H, t, $^4J_{HH}$ = 1.8 Hz, H_a), 7.49 (2H, d, $^3J_{HH}$ = 8.0 Hz, H_f), 7.47 (2H, d, $^4J_{HH}$ = 1.8 Hz, H_b), 4.66 (4H, m, H_c + H_d), 4.50 (2H, s, H_g), 1.31 (18H, s, H_h).

^{13}C NMR (126 MHz, *d*$_6$-acetone): 151.7 (Ar***C***), 137.6 (Ar***C***), 131.0 (Ar***C***), 130.7 (Ar***C***H), 130.5 (Ar***C***), 129.0 (Ar***C***H), 124.2 (Ar***C***H), 123.6 (Ar***C***H), 53.6 (***C***H_2NH_2), 52.5 (NH_2***C***H_2), 51.4 (***C***H_2N_3), 34.6 (***C***CH_3), 30.7 (C***C***H_3).

^{19}F NMR (470 MHz, *d*$_6$-acetone): -72.5 (d, $^1J_{FP}$ = 706 Hz).

^{31}P NMR (202 MHz, *d*$_6$-acetone): -144.4 (sept., $^1J_{PF}$ = 706 Hz).

HRMS (ESI): Calc. ($C_{23}H_{33}N_4$) m/z = 365.2700 $[M]^+$; Found m/z = 365.2711.

3,5-Tertbutyl-ethynylbenzene **10**

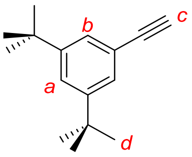

12 was synthesized via a new route using the Corey-Fuchs reaction:[6]

Triphenylphosphine (3.61 g, 13.8 mmol) and carbon tetrabromide (2.28 g, 6.88 mmol) were dissolved in anhydrous dichloromethane (10 mL) and stirred at 0 °C for 30 minutes. 3,5-tert-butylbenzaldehyde (750 mg, 3.44 mmol) was dissolved separately in dichloromethane (10 mL) and added dropwise to the chilled reaction over 5 minutes. After stirring at 0 °C for a further hour, the reaction was washed

with 5 M $CuSO_4$ (30 mL) and the aqueous and organic layers separated. The aqueous layer was back-extracted with chloroform (20 mL) and the combined organics was washed with brine (20 mL) and dried with $MgSO_4$. The crude yellow oil obtained after solvent removal was purified using a short silica plug (eluent: dichloromethane) to give the 1,1-dibromoalkene intermediate as a colourless oil.

Subsequently, the 1,1-dibromoalkene intermediate was dissolved in anhydrous diethyl ether (18 mL) and chilled to - 78 ^{o}C in a dry ice/ acetone bath under N_2. *n*-Butyllithium (2.5 M in hexanes) (3.4 mL, 8.60 mmol) was then added dropwise to the vigorously-stirred reaction and left to react for 30 minutes before warming up to ambient temperature and stirred for a further hour. The reaction was quenched by the slow addition of water (10 mL) under N_2. After separating the aqueous and organic layers, the aqueous layer was extracted with diethyl ether (4 x 15 mL) and the combined organics dried with $MgSO_4$. The solvent was removed *in vacuo* to afford the target product as a white solid in excellent purity (552 mg, 75 %) and hence no further purification was necessary.

^{1}H NMR (400 MHz, $CDCl_3$): 7.42 (1H, t, $^4J_{HH}$ = 1.8 Hz, H_a), 7.36 (2H, d, $^4J_{HH}$ = 1.8 Hz, H_b), 3.03 (1H, s, H_c), 1.32 (19H, s, H_d). ^{1}H NMR spectrum matches that previously reported.[7]

<u>Synthesis of rotaxane **2**</u>

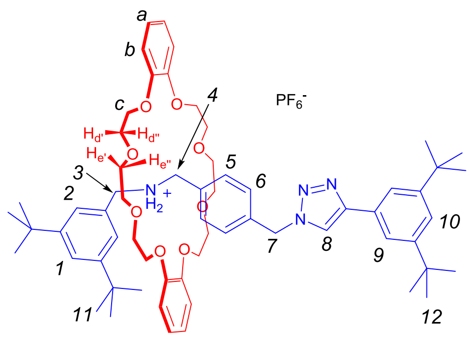

Synthesis of copper(I)-acetylide (*using a reported method*):[8]

A solution of alkyne **10** (180 mg, 0.84 mmol) in ethanol (2.5 mL) was added to a vigorously-stirred solution of CuI (400 mg, 2.1 mmol) in 38 % aqueous ammonia (5 mL) at room temperature to form a green suspension. After stirring for 1 hour, the suspension was filtered and the orange residue washed with water (c.a. 100 mL) till the filtrate turned colourless. The residue was air-dried to afford the target copper(I)-acetylide as an air-stable bright orange solid (200 mg, 86 %) which was used without further purification.

Synthesis of rotaxane

Dibenzo[24]crown[8] macrocycle (180 mg, 0.40 mmol) and azide **9** (102 mg, 0.20 mmol) were dissolved in anhydrous dichloromethane (0.5 mL) and stirred for 15 minutes before the copper(I)-acetylide (55 mg, 0.20 mmol) was added portionwise. The orange suspension was stirred for 3 days under N_2, over which it cleared to form a slightly cloudy orange solution. The reaction was diluted with chloroform (30 mL) and washed with 0.02 M ethylenediaminetetraacetic acid (EDTA) in 1 M aqueous ammonia (2 x 20 mL). The aqueous layer was back-extracted with chloroform (2 x 10 mL) and the combined organics were dried with $MgSO_4$. Silica gel preparatory thin layer chromatography (eluent: 2 % methanol in dichloromethane *v/v*) afforded the target rotaxane contaminated with small quantities of unreacted DB24C8 macrocycle. After removing the product from silica gel by stirring with 10 % methanol in dichloromethane *v/v* for 1 hour, the pale beige solid was further purified by size-exclusion column chromatography (eluent: chloroform) to afford the rotaxane in excellent purity. Anion exchange was performed by washing a chloroform solution of the purified rotaxane with 0.2 M NH_4PF_6 (aq.) (8 x 15 mL) then water (20 mL). After drying with $MgSO_4$, solvent removal *in vacuo* and drying in a vacuum dessicator over P_2O_5 for 3 days afforded the target rotaxane as a white solid (128 mg, 55 %). The rotaxane was stored in a N_2-filled glovebox in between usage.

^{1}H NMR (500 MHz, d_6-acetone): 8.36 (1H, s, H_8), 7.78 (2H, d, $^4J_{HH}$ = 1.8 Hz, H_9), 7.75 (2H, br. s., $R_2NH_2^+$), 7.50 (1H, t, $^4J_{HH}$ = 1.8 Hz, H_{10}), 7.47 (1H, t, $^4J_{HH}$ = 1.9 Hz, H_1), 7.46 (2H, d, $^4J_{HH}$ = 1.9 Hz,

H_2), 7.30 (2H, d, $^3J_{HH}$ = 8.0 Hz, H_5), 6.98 (2H, d, $^3J_{HH}$ = 8.0 Hz, H_6), 6.84-6.89 (8H, m, H_{a+b}), 5.46 (2H, s, H_7), 4.87-4.90 (2H, m, H_3), 4.75-4.78 (2H, m, H_4), 4.16-4.18 (8H, m, H_c), 3.89-3.93 (4H, m, $H_{d''}$), 3.79-3.84 (4H, m, $H_{d'}$), 3.73-3.77 (4H, m, $H_{e''}$), 3.57-3.60 (4H, m, $H_{e'}$), 1.36 (18H, s, H_{12}), 1.23 (18H, s, H_{11}).

^{13}C NMR (126 MHz, d_6-acetone): 151.3, 151.2, 148.2, 147.6, 137.0, 131.8, 131.7, 130.6, 130.0, 127.6, 123.8, 123.3, 121.9, 121.2, 120.8, 119.7, 112.5, 70.5, 70.2, 67.9, 52.9, 52.8, 52.2, 34.6, 34.5, 30.9, 30.8.

^{19}F NMR (470 MHz, d_6-acetone): -72.2 (d, $^1J_{FP}$ = 706 Hz).

^{31}P NMR (202 MHz, d_6-acetone): -144.4 (sept., $^1J_{PF}$ = 706 Hz).

HRMS (ESI): Calc. ($C_{63}H_{87}N_4O_8$) m/z = 1027.6518 $[M]^+$; Found m/z = 1027.6493.

S2.3 Synthesis of Rotaxane 3

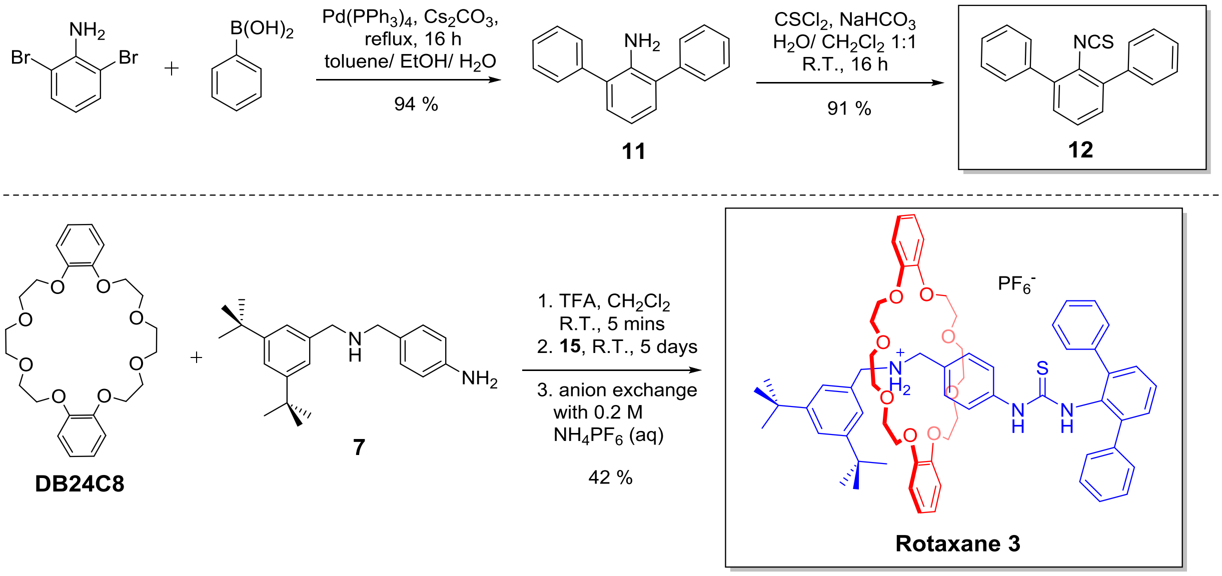

Scheme 3. Synthesis of rotaxane **3**.

Compounds **11**[9] and **12**[10] were synthesised as reported.

<u>Synthesis of rotaxane **3**</u>

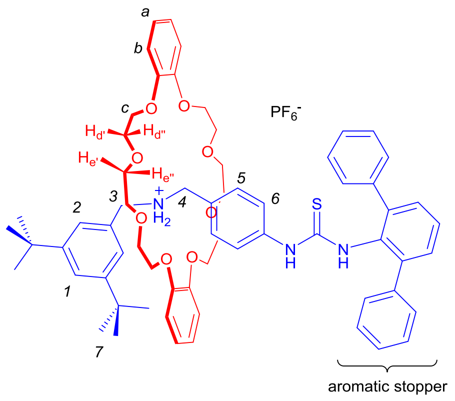

The synthesis was analogous to that of rotaxane **1** (Section S2.3). To a solution of aniline axle precursor **7** (65 mg, 0.20 mmol) and dibenzo[24]crown[8] macrocycle (135 mg, 0.30 mmol) in

anhydrous dichloromethane (0.5 mL) was added neat trifluoroacetic acid (15 µL, 0.20 mmol. After stirring for 15 minutes, isothiocyanate **12** (180 mg, 0.69 mmol) was added portionwise and the reaction stirred at room temperature for 7 days. The reaction was diluted with dichloromethane (30 mL) and washed successively with saturated aqueous sodium bicarbonate (20 mL) and water (20 mL) then dried with $MgSO_4$. The crude solid obtained after rotary evaporation of the solvent, was purified by silica preparatory thin layer chromatography (eluent: 4 % methanol in dichloromethane *v/v*). After extracting the purified rotaxane from the silica gel using 10 % methanol in dichloromethane, the rotaxane was further purified by size-exclusion chromatography (eluent: chloroform).

Anion exchange was performed by dissolving the purified rotaxane in chloroform (30 mL) and washing successively with 0.2 M NH_4PF_6 (aq.) (8 x 15 mL) then water (20 mL). The organic layer was dried with $MgSO_4$ and solvent was removed to afford the target rotaxane as a pale beige solid (65 mg, 27 %). The rotaxane was dried thoroughly *in vacuo* and in a vacuum dessicator over P_2O_5 for at least 3 days, and stored in a N_2-filled glovebox in between usage.

^{1}H NMR (500 MHz, *d*$_6$-acetone): 7.66 (2H, br. s., $R_2NH_2^+$), 7.58 (2H, d, $^4J_{HH}$ = 1.5 Hz, H_2), 7.30-7.57 (16H, complex m, H_1+ H_6 + aromatic stoppers), 7.08 (2H, d, $^3J_{HH}$ = 7.4 Hz, H_5), 6.90-6,97 (4H, m, H_a), 6.80-6.83 (4H, m, H_b), 4.84-4.86 (2H, m, H_3), 4.60-4.64 (2H, m, H_4), 4.12-4.21 (8H, m, H_c), 3.87-3.90 (4H, m, $H_{d''}$), 3.78-3.82 (4H, m, $H_{d'}$), 3.63-3.71 (4H, m, $H_{e''}$), 3.52-3.57 (4H, m, $H_{e'}$), 1.22 (18H, s, H_7).

^{13}C NMR (126 MHz, *d*$_6$-acetone): 152.1, 150.3, 148.6, 142.6, 132.7, 130.8, 130.1, 130.0, 129.0, 128.9, 128.3, 127.9, 124.8, 124.2, 1212.3, 122.2, 115.6, 113.6, 113.5, 113.4, 71.4, 71.1, 69.0, 53.7, 53.6, 53.2, 35.5, 31.7.

^{19}F NMR (470 MHz, *d*$_6$-acetone): -72.2 (d, $^1J_{FP}$ = 706 Hz).

^{31}P NMR (202 MHz, *d*$_6$-acetone): -144.4 (sept., $^1J_{PF}$ = 706 Hz).

HRMS (ESI): Calc. ($C_{65}H_{78}N_3O_8\,^{32}S$) m/z = 1060.5504 $[M]^+$; Found m/z = 1060.5498.

S2.4 Synthesis of Acyclic Catalyst 4

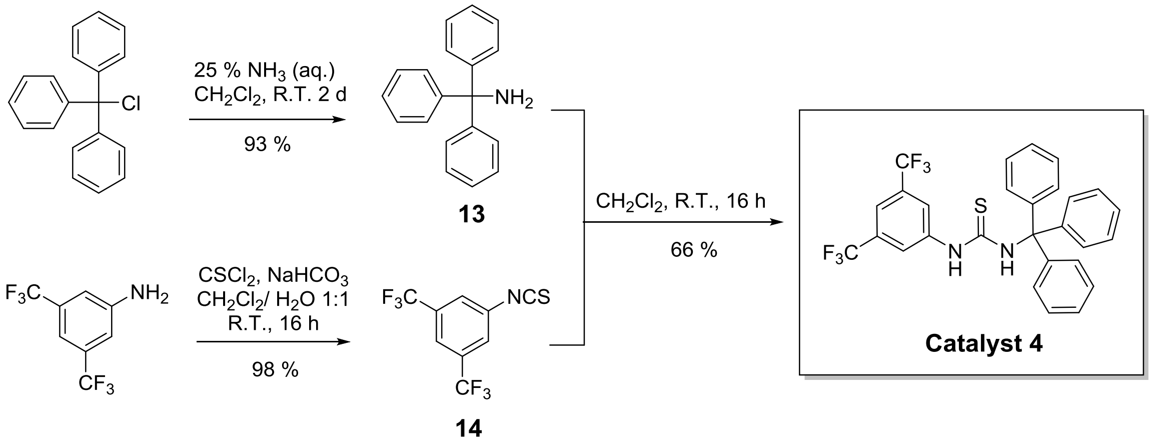

Scheme 4. Synthesis of acyclic catalyst **4**.

Compounds **13**[11] and **14**[12] were synthesized as reported.

Catalyst **4**

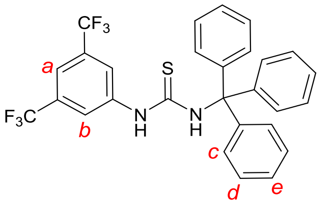

Equimolar quantities of tritylamine **13** (210 mg, 0.81 mmol) and isothiocyanate **14** (220 mg, 0.81 mmol) were dissolved in anhydrous dichloromethane (2 mL) and stirred overnight at room temperature. Thereafter, silica gel column chromatography (eluent: hexane/ dichloromethane 1:1 *v/v*) afforded the target compound as a white solid (284 mg, 66 %).

^{1}H NMR (500 MHz, d_6-acetone): 8.17 (2H, br. s, TU-H), 7.93 (2H, br. s, H_b), 7.68 (1H, s, H_a), 7.45 (6H, d, $^3J_{HH}$ = 7.5 Hz, H_c), 7.39 (6H, m, H_d), 7.32 (3H, m, H_e).

^{13}C NMR (126 MHz, d_6-acetone): 182.1 (NH***C***SNH), 144.7 (Ar***C***), 142.5 (Ar***C***), 131.7 (quart., $^2J_{CF}$ = 34 Hz, ***C***CF_3), 130.0 (Ar***C***H), 129.2 (Ar***C***H), 128.4 (Ar***C***H), 124.3 (quart, $^1J_{CF}$ = 273 Hz, ***C***F_3), 124.3 (m, Ar***C***), 118.4 (m, Ar***C***), 74.1 (***C***Ph_3).

^{19}F NMR (470 MHz, d_6-acetone): -63.6.

HRMS (ESI): Calc. ($C_{28}H_{21}N_2F_6{}^{32}S$) m/z = 531.1324 $[M+H]^+$; Found m/z = 531.1326.

S2.5 Synthesis of Thiourea-containing Free Axle

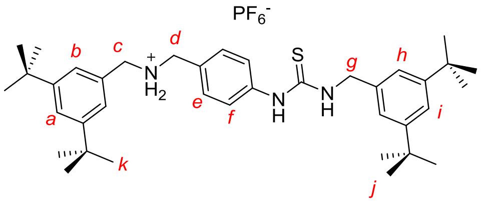

The neutral axle (55 mg) was isolated as a side-product from the synthesis of rotaxane **1** (Section S2.2). The protonated axle was obtained by dissolving the neutral axle in a minimum quantity of acetone and adding to 3 M HCl (50 mL). The white precipitate was filtered and re-dissolved in acetone (c.a. 1 mL) and re-precipitated in 0.25 M NH_4PF_6 (aq.). Vacuum filtration of the suspension was followed by drying over P_2O_5 in a vacuum dessicator for 3 days afforded the product as a white solid (52 mg, 75 %).

^{1}H NMR (500 MHz, d_6-acetone): 9.05 (1H, br. s, TU-H), 8.61 (2H, br.s, $R_2NH_2^+$), 7.65 (3H, m, TU-H + H_f), 7.57 (1H, d, $^4J_{HH}$ = 1.1 Hz, H_a), 7.50 (2H, d, $^3J_{HH}$ = 8.2 Hz, H_e), 7.40 (1H, d, $^4J_{HH}$ = 1.0 Hz, H_i), 7.29 (2H, d, $^4J_{HH}$ = 1.0 Hz, H_h), 4.83 (2H, s, H_g), 4.64 (2H, s, H_c), 4.61 (2H, s, H_d), 1.31 (36H, s, H_j + H_k).

^{13}C NMR (126 MHz, d_6-acetone): 181.6 (NH***C***SNH), 151.7 (Ar***C***), 150.8 (Ar***C***), 141.0 (Ar***C***), 137.6 (Ar***C***NH), 130.7 (Ar***C***H), 130.5 (Ar***C***), 126.6 (Ar***C***), 124.1 (Ar***C***H), 123.5 (Ar***C***H), 122.1 (Ar***C***H), 121.1 (Ar***C***H), 52.3 (TU-***C***H_2), 51.5 (***C***$H_2NH_2^+$), 48.5 (NH_2^+***C***H_2), 34.6 (***C***$(CH_3)_3$), 34.5 (***C***$(CH_3)_3$), 30.8 (C(***C***$H_3)_3$), 30.7 (C(***C***$H_3)_3$).

^{19}F NMR (470 MHz, d_6-acetone): -72.6 (d, $^1J_{FP}$ = 706 Hz).

^{31}P NMR (202 MHz, d_6-acetone): -144.2 (sept., $^1J_{PF}$ = 706 Hz).

HRMS (ESI): Calc. ($C_{38}H_{56}N_3{}^{32}S$) m/z = 586.4190 $[M]^+$; Found m/z = 586.4190.

S2.6 Synthesis of Triazole-containing Free Axle

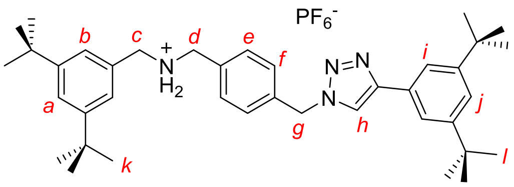

The neutral axle (40 mg) was isolated as a side-product from the synthesis of rotaxane **2** (Section S2.3) and protonated followed by anion exchange as described above for the thiourea-containing free axle. The target compound was isolated as a white solid (37 mg, 74 %) after drying over P_2O_5 in a vacuum dessicator for 3 days.

^{1}H NMR (500 MHz, d_6-acetone): 8.95 (2H, br. s, $R_2NH_2^+$), 8.49 (1H, s, H_h), 7.77 (2H, d, ^4J = 1.4 Hz, H_i), 7.61 (2H, d, ^3J = 7.8 Hz, H_e), 7.54 (1H, s, H_j), 7.44-7.48 (5H, m, H_a + H_b + H_f), 5.70 (2H, s, H_g), 4.56 (4H, m, H_c + H_d), 1.35 (18H, s, H_l), 1.29 (18H, s, H_k).

^{13}C NMR (126 MHz, d_6-acetone): 151.6 (Ar***C***), 151.2 (Ar***C***), 148.3 (Ar***C***), 137.8 (Ar***C***), 131.2 (Ar***C***), 130.8 (Ar***C***H), 130.4 (Ar***C***), 128.5 (Ar***C***H), 124.3 (Ar***C***H), 123.4 (Ar***C***H), 121.9 (Ar***C***H), 120.9 (triazole-Ar***C***H), 119.8 (Ar***C***H), 52.9 (***C***H_2-triazole), 52.1 (***C***$H_2NH_2^+$), 51.1 (NH_2^+***C***H_2), 34.7 (***C***$(CH_3)_3$), 34.6 (***C***$(CH_3)_3$), 30.9 (C(***C***$H_3)_3$), 30.8 (C(***C***$H_3)_3$).

^{19}F NMR (470 MHz, d_6-acetone): -72.3 (d, $^1J_{FP}$ = 706 Hz).

^{31}P NMR (202 MHz, d_6-acetone): -144.2 (sept., $^1J_{PF}$ = 706 Hz).

HRMS (ESI): Calc. ($C_{39}H_{55}N_4$) m/z = 579.4421 $[M]^+$; Found m/z = 579.4418.

S2.7 Typical polymerization procedure with rotaxanes 1-3

All the polymerizations were set up (except low temperature polymerization) in a N_2- filled glove box. In a typical reaction, the polymerization vessel was charged with *rac*-lactide (72 mg, 0.5 mmol) which was then dissolved in dry THF (0.3 mL). The catalyst mixture was prepared in a separate vessel by dissolving 0.010 mmol of the protonated rotaxane in 0.195 mL of a stock solution of $KN(SiMe_3)_2$ in THF (10 mg/ mL), stirring for 3 minutes, then adding a 5 µL of a 2 M stock solution of benzyl alcohol (BnOH) in THF to it. After stirring for a further 2 minutes, the activated catalyst mixture was injected into the solution of monomer such that [LA] = 1.0 M and [rotaxane/ $KN(SiMe_3)_2$/ BnOH] = 20 mM. The polymerization/ aliquots were quenched at the desired time with a solution of excess benzoic acid in dichloromethane and the solvent evaporated under a stream of N_2. The crude products were analysed by ^{1}H NMR spectroscopy (^{1}H{^{1}H} NMR where applicable) in $CDCl_3$ and GPC in THF. Percent lactide conversions were calculated from the ratio of integrals of the methine resonances of the monomer (4.96 - 5.04 ppm) and polymer (5.10 - 5.22 ppm). The tacticity (P_i) was determined by integrating the tetrad peaks in the deconvoluted ^{1}H{^{1}H} NMR spectra and comparison of peak intensities against those predicted by Bernoullian statistics (*vide infra*).[13] Each sample for GPC analysis was prepared by dissolving the crude polymer in HPLC grade THF (10 mg of polymer/ mL) before filtering the clear solution through a 13 mm syringe filter with 0.2 µm PTFE membrane (VWR International).

When polymerization was performed at elevated temperatures (323 K), the polymerizations were prepared in the glove box as described and the vessel sealed with an airtight lid before removing from the glovebox and heated in an oil bath for the desired duration. The reaction was quenched using excess benzoic acid.

Polymer purification: The crude polymer mixture was dissolved in chloroform (~1 mL) and added dropwise to methanol (~10 mL) to precipitate the polymer. The mixture was filtered and the polymer was then air-dried. The polymer was re-dissolved in chloroform (~ 1 mL) and rapidly filtered through a

small pad of silica (washed with excess chloroform ~ 5 mL). The solvent was removed *in vacuo* and dried under vacuum at 40 ^{o}C till constant weight was obtained.

Determination of stereocontrol degree of polymers

The microstructure of a polymer was determined from its $^1H\{^1H\}$ NMR spectrum (see Figures S34-40). The methine region of each $^1H\{^1H\}$ NMR spectra exhibited five different peaks arising from each tetrad possibility and reflect the stereochemistry along the polymer chain. The deconvoluted spectrum was analysed with MestReNova v.11.0.2 using the Global Spectral Deconvolution (GSD) algorithm (level 2) with 5 fitting cycles, and the peak areas of the deconvoluted peaks were used to calculate the normalised tetrad integrals. The P_i values, reflecting the probability of isotactic enchainment, were identified from each of tetrad integrals using the following Bernoullian statistical equations:[13]

$$[iii] = P_i^2 + P_iP_s/2 \quad \textbf{(1)}$$

$$[iis] = P_sP_i/2 \quad \textbf{(2)}$$

$$[sii] = P_sP_i/2 \quad \textbf{(3)}$$

$$[sis] = P_s^2/2 \quad \textbf{(4)}$$

$$[isi] = (P_s^2 + P_sP_i)/2 \quad \textbf{(5)}$$

At least three P_i values for each tetrad integral calculated using the above equations were used to determine the average P_i values (Table 1, manuscript). The errors associated with each P_i value was obtained by calculating the standard error of the mean, which is defined by the following equation, where σ = standard deviation and N = no. of values used (i.e. N = 3 if 3 P_i values were used):

$$\text{Standard error} = \frac{\sigma}{\sqrt{N}}$$

S2.8 Spectral Characterisation of Catalysts

Rotaxane **1**

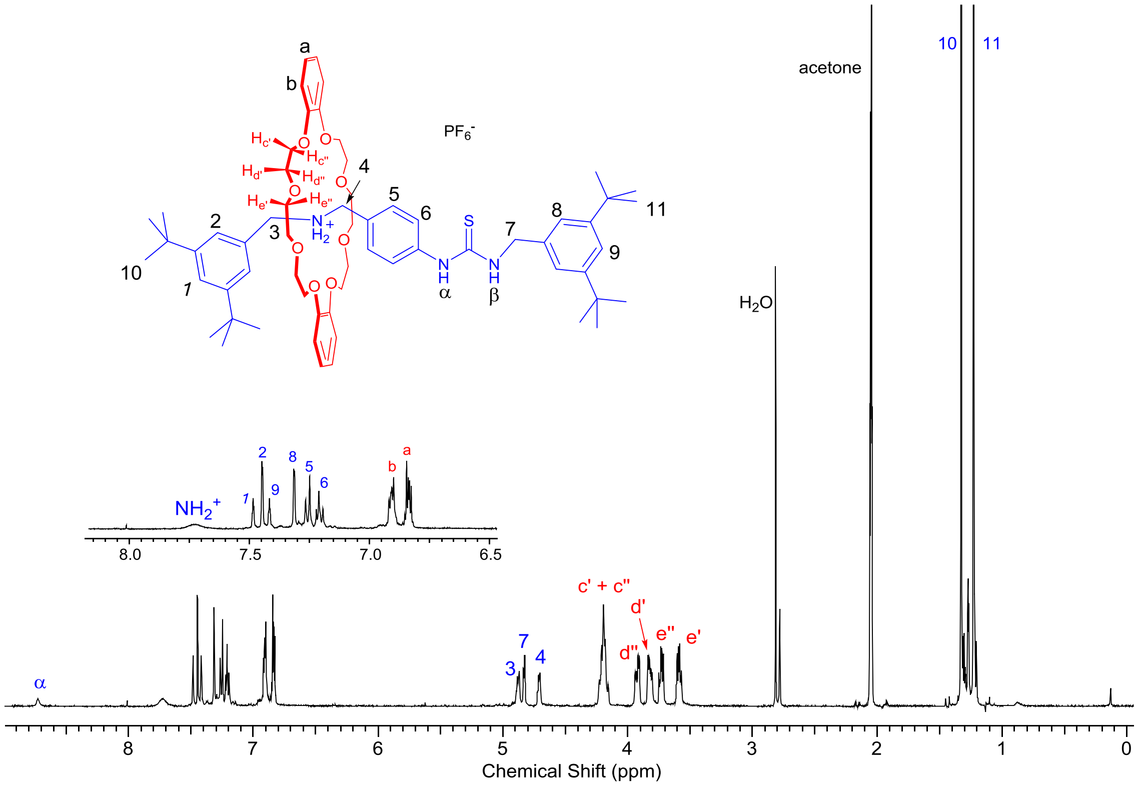

Figure S1. ^{1}H NMR of rotaxane **1** in d$_6$-acetone.

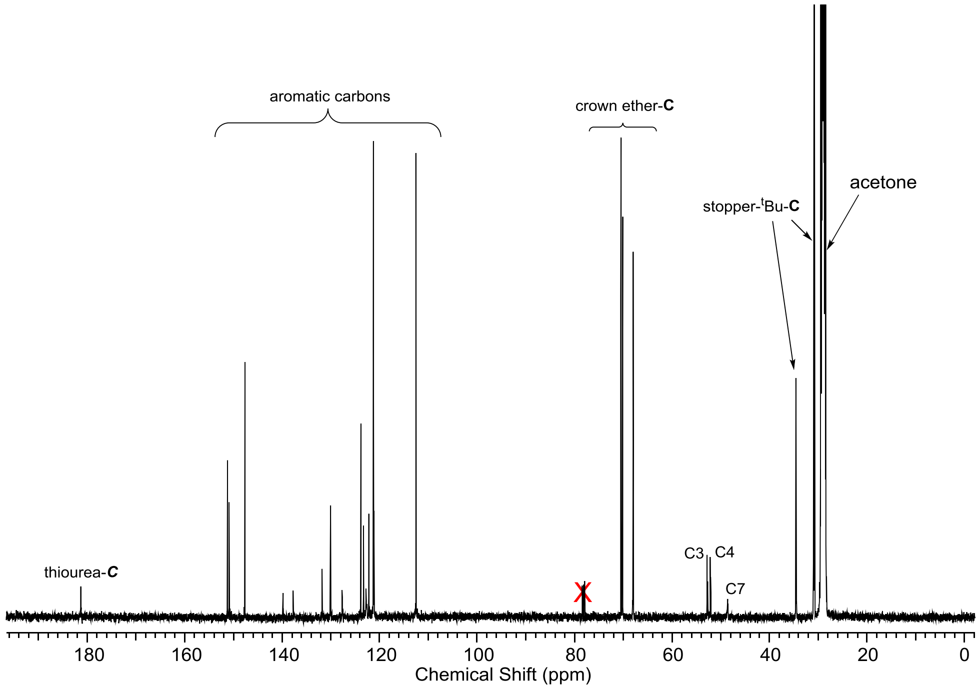

Figure S2. ^{13}C NMR of rotaxane **1** in d$_6$-acetone (assignments follow those in Fig S1).

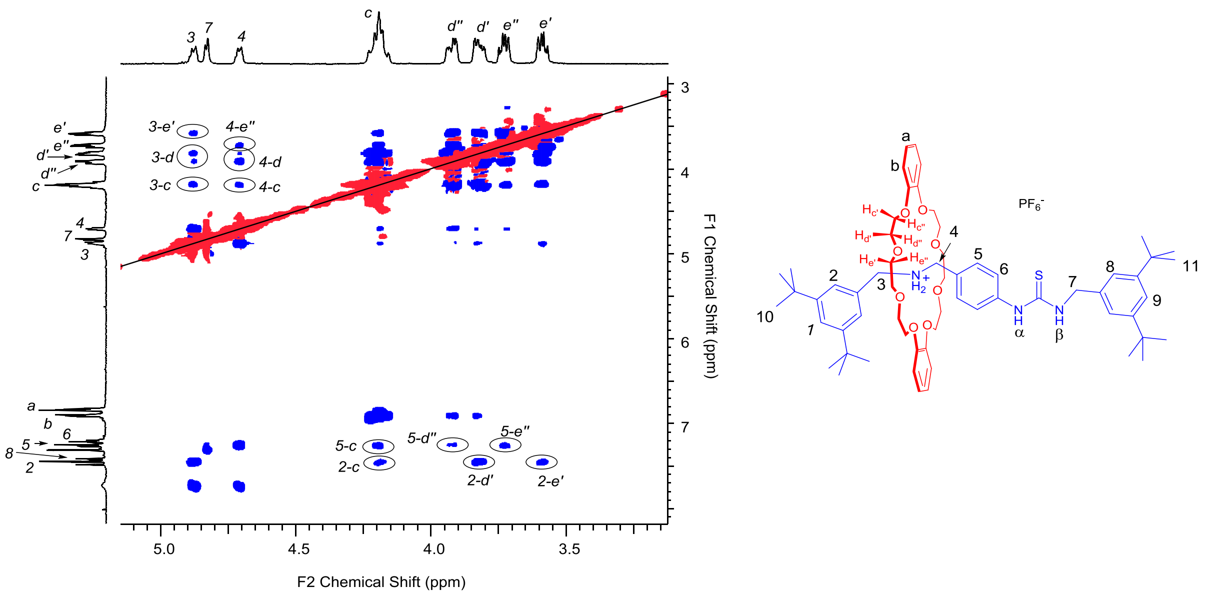

Figure S3. Two-dimensional ^1H-^1H ROESY NMR spectrum of rotaxane **1** in d_6-acetone. Cross-peaks arising from through-space interactions between macrocycle and axle protons are circled.

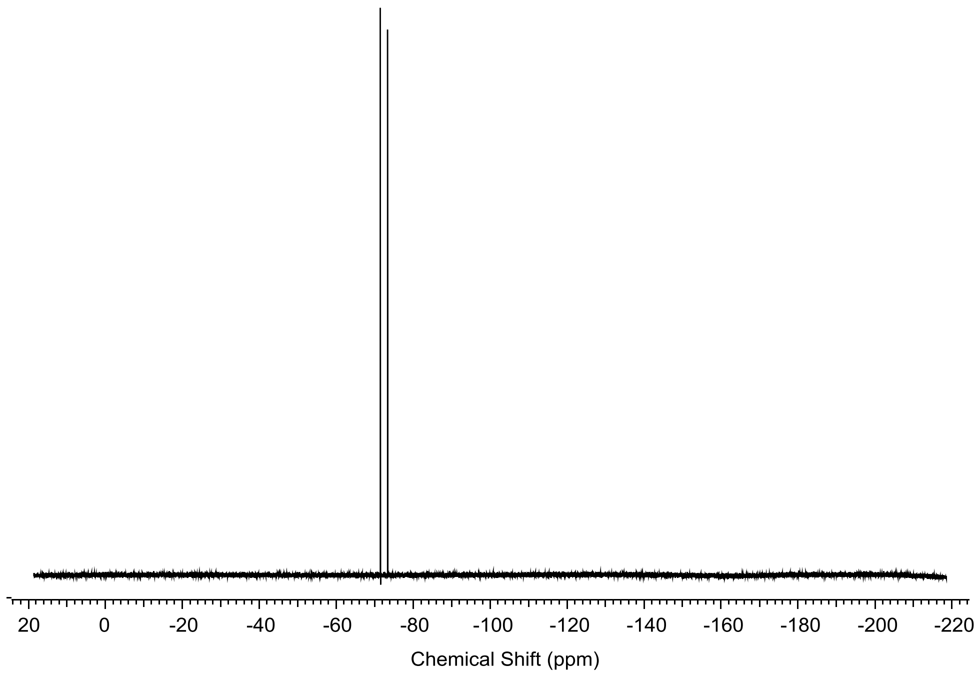

Figure S4. ^{19}F NMR of rotaxane **1** in d_6-acetone.

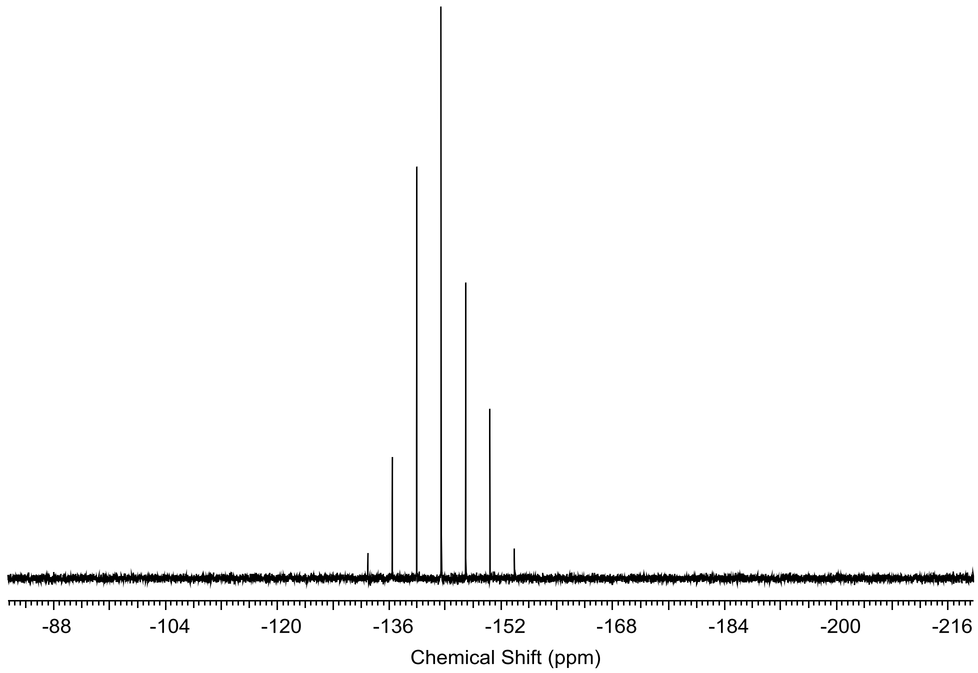

Figure S5. ^{31}P NMR of rotaxane **1** in d_6-acetone.

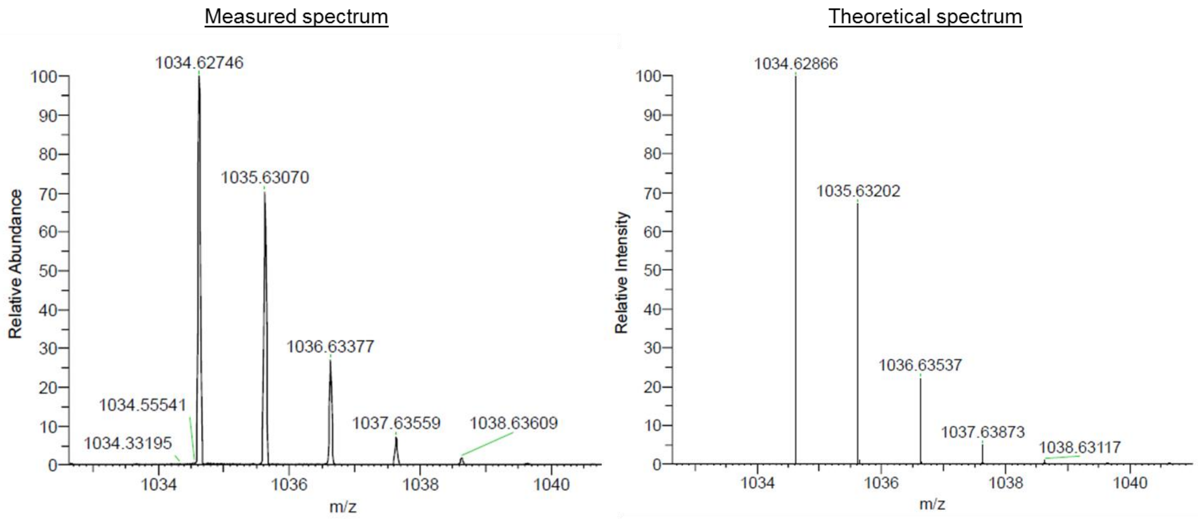

Figure S6. High-resolution ESI-MS characterization (positive mode) of rotaxane **1** (left- measured spectrum; right- theoretical spectrum).

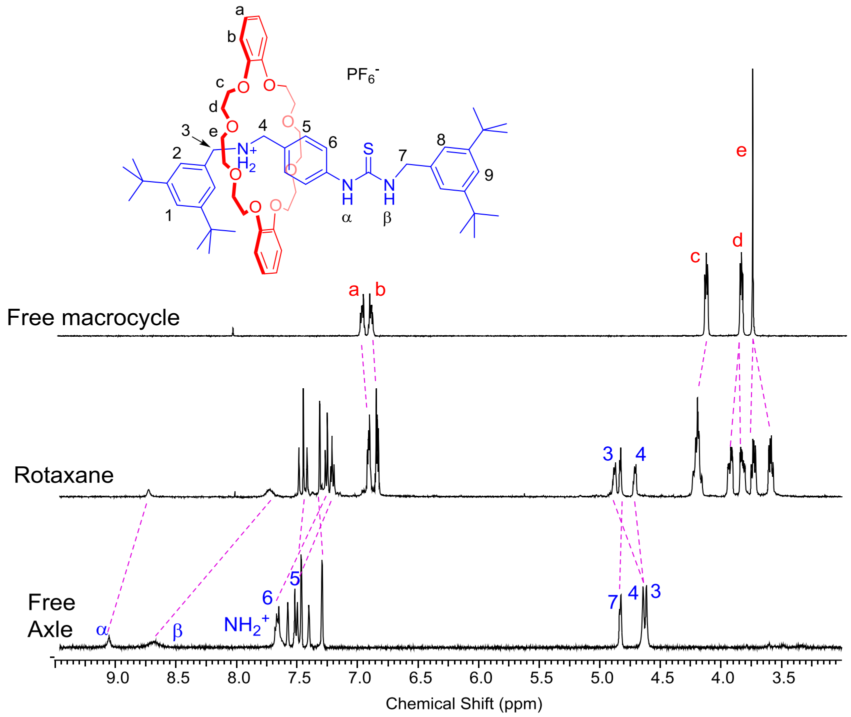

Figure S7. Stacked partial ^{1}H NMR spectra of the non-interlocked crown ether macrocycle, rotaxane **1** and free protonated axle (PF_6^- salt) in d_6-acetone to show the changes in chemical environments of various proton signals upon rotaxane formation.

<u>Rotaxane **2**</u>

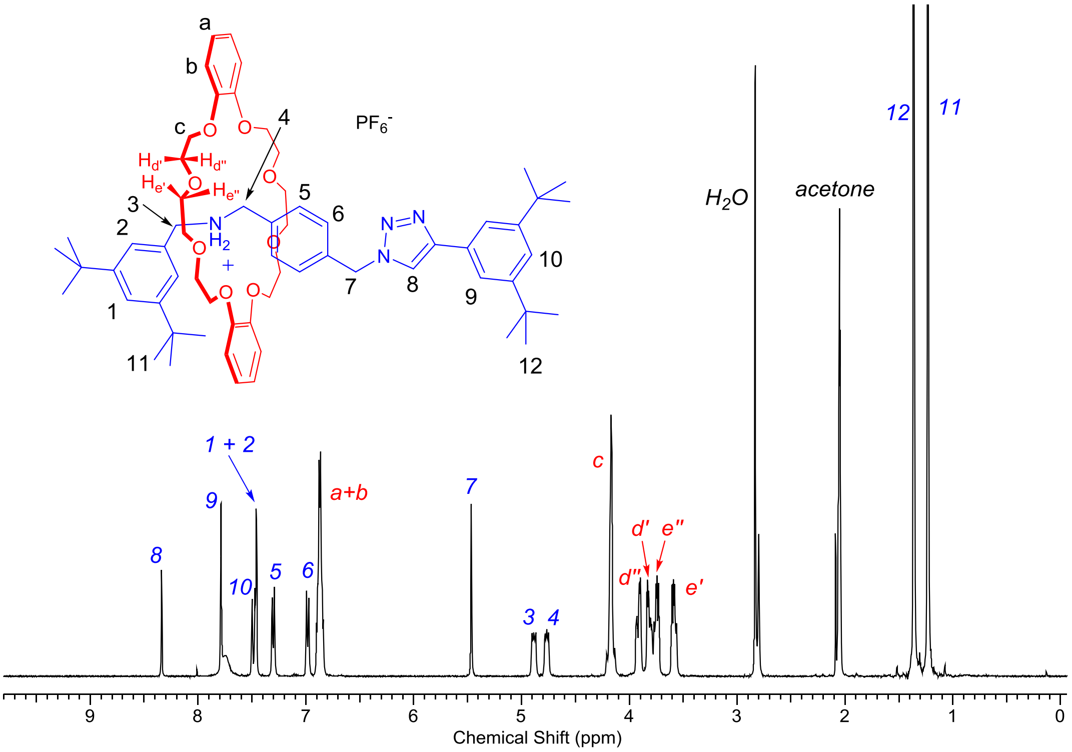

Figure S8. ^{1}H NMR of rotaxane **2** in d_6-acetone.

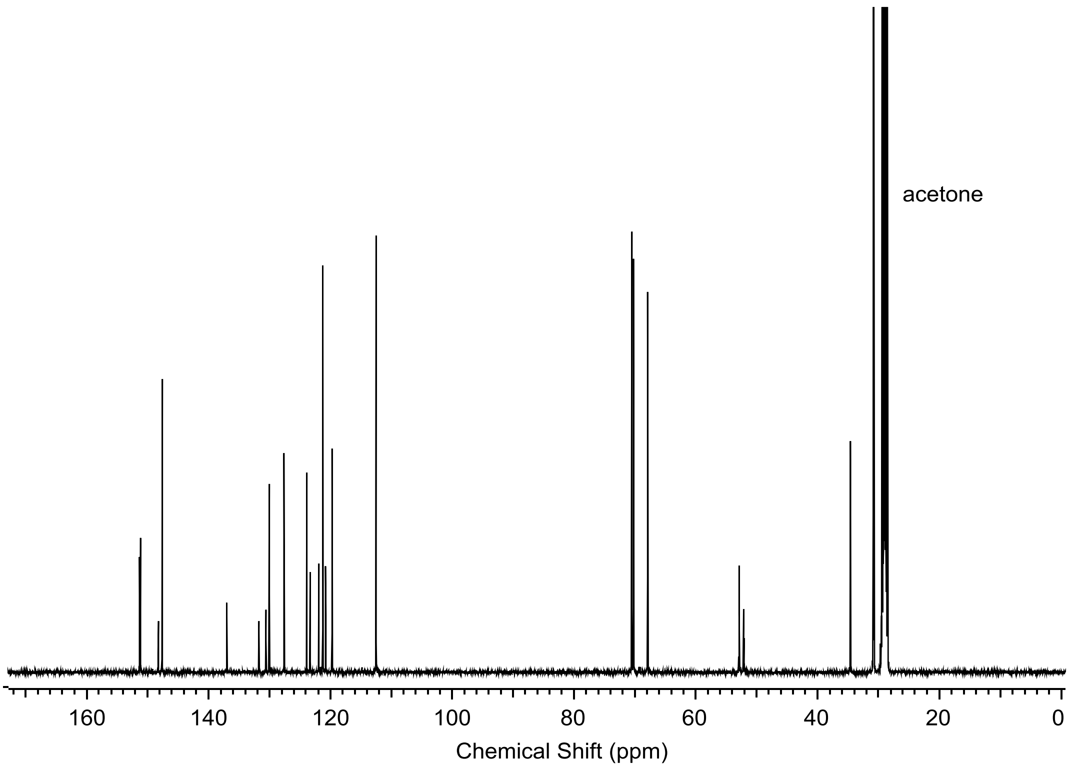

Figure S9. ^{13}C NMR of rotaxane **2** in d6-acetone.

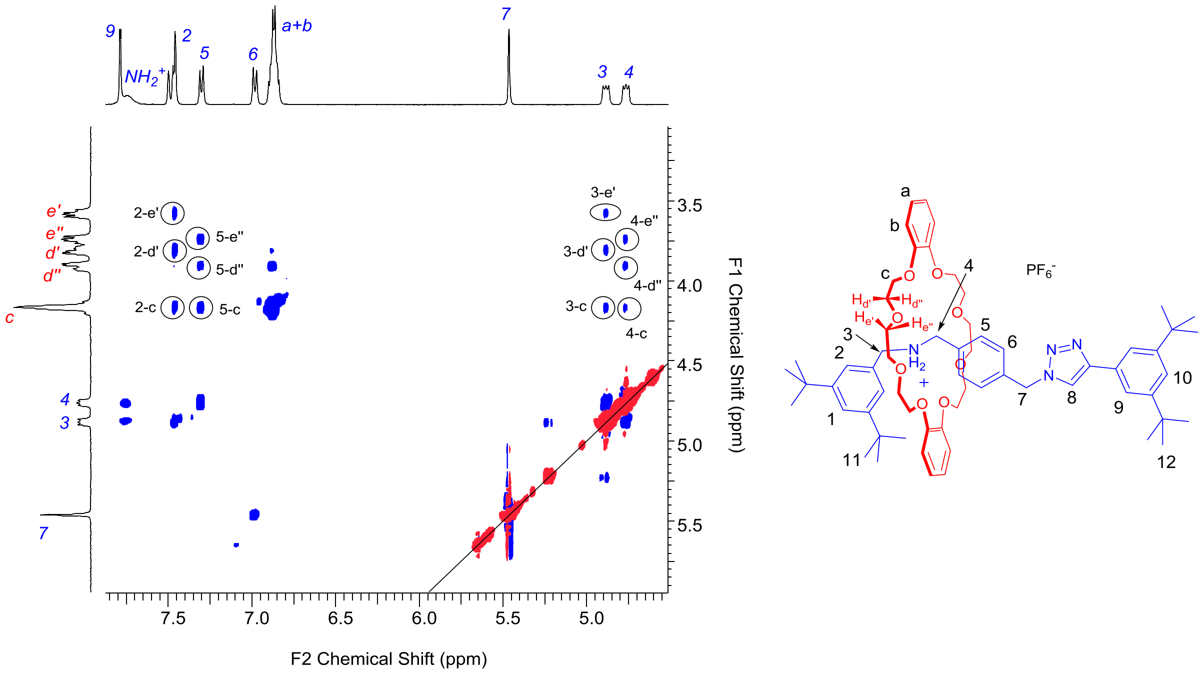

Figure S10. Two-dimensional ^{1}H-^{1}H ROESY NMR spectrum of rotaxane **2** in d6-acetone. Cross-peaks arising from through-space interactions between macrocycle and axle protons are circled.

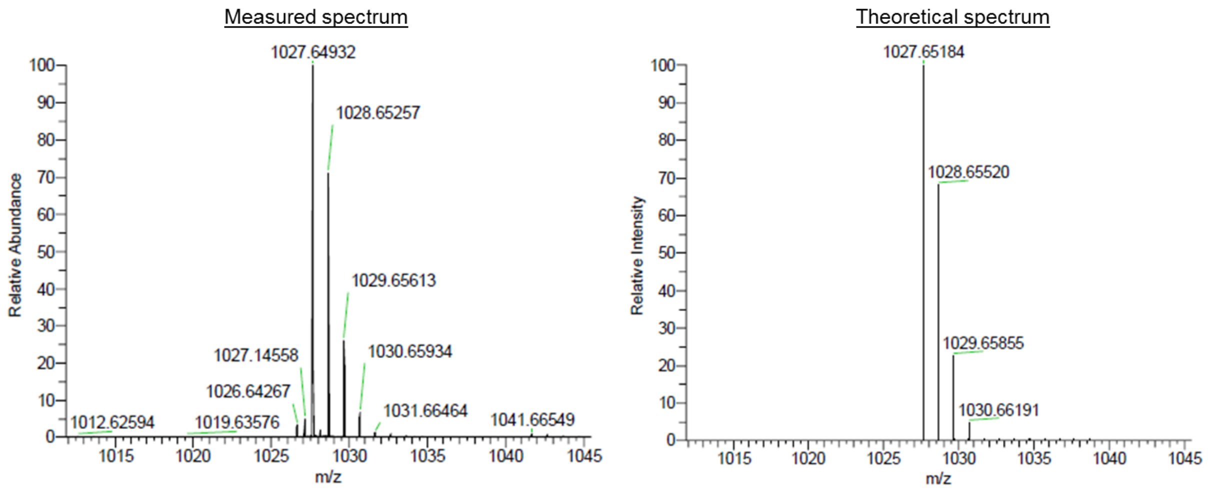

Figure S11. High-resolution ESI-MS characterization (positive mode) of rotaxane **2** (left- measured spectrum; right- theoretical spectrum).

Rotaxane **3**

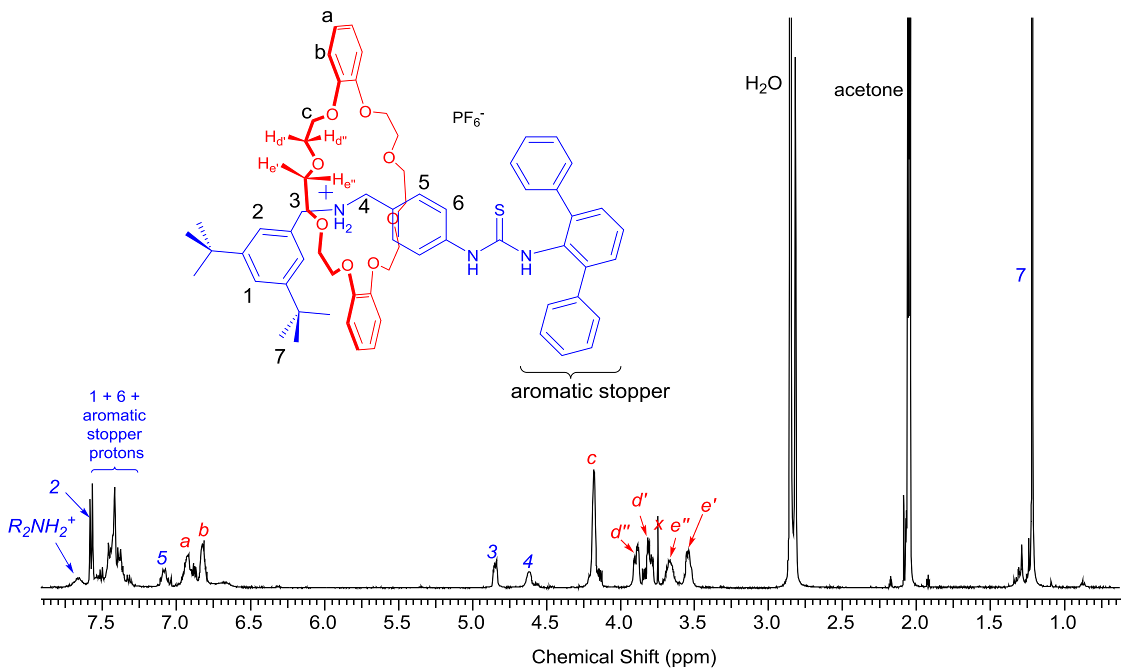

Figure S12. ^{1}H NMR of rotaxane **3** in d6-acetone.

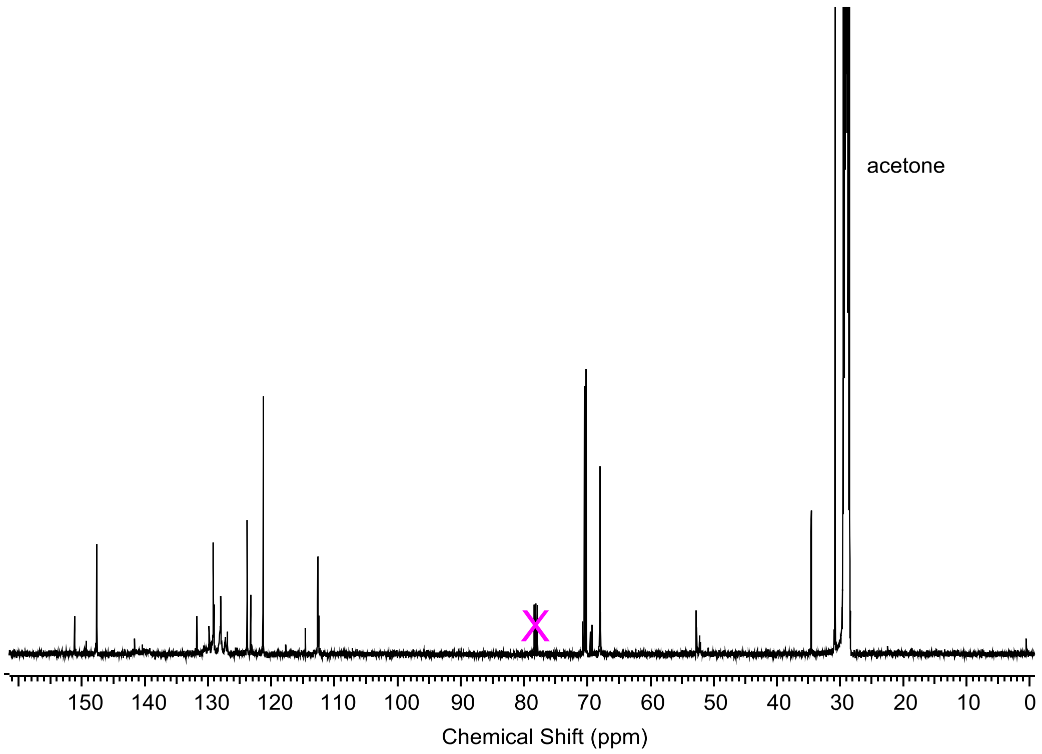

Figure S13. ^{13}C NMR of rotaxane **3** in d6-acetone.

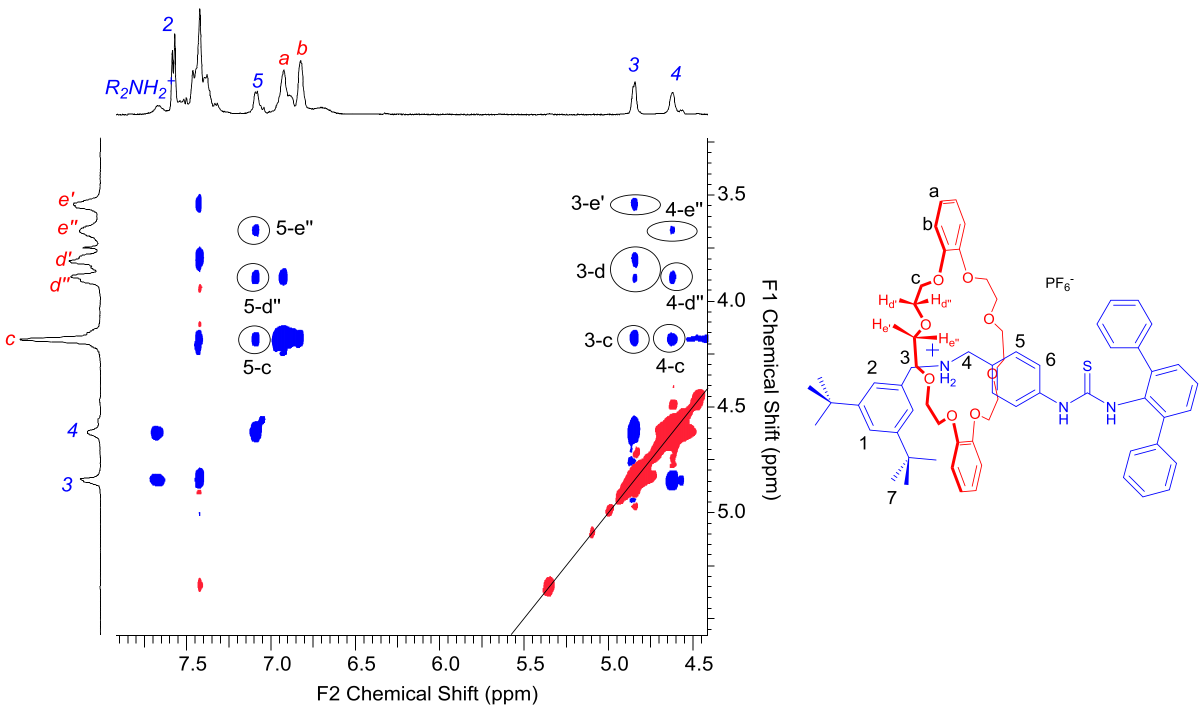

Figure S14. Two-dimensional ^1H-^1H ROESY NMR spectrum of rotaxane **3** in d6-acetone. Cross-peaks arising from through-space interactions between macrocycle and axle protons are circled.

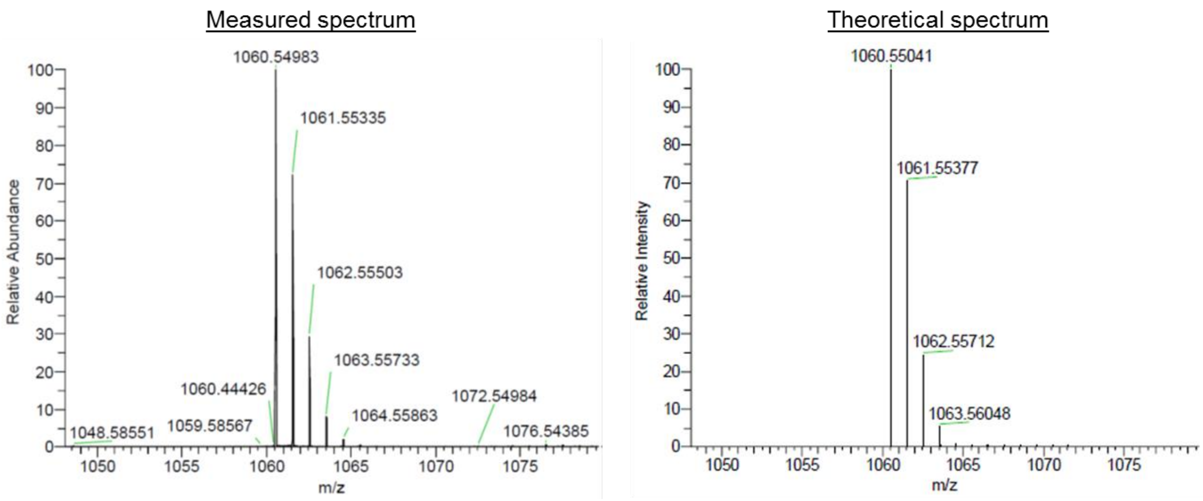

Figure S15. High-resolution ESI-MS characterization (positive mode) of rotaxane **3** (left- measured spectrum; right- theoretical spectrum).

Acyclic Catalyst **4**

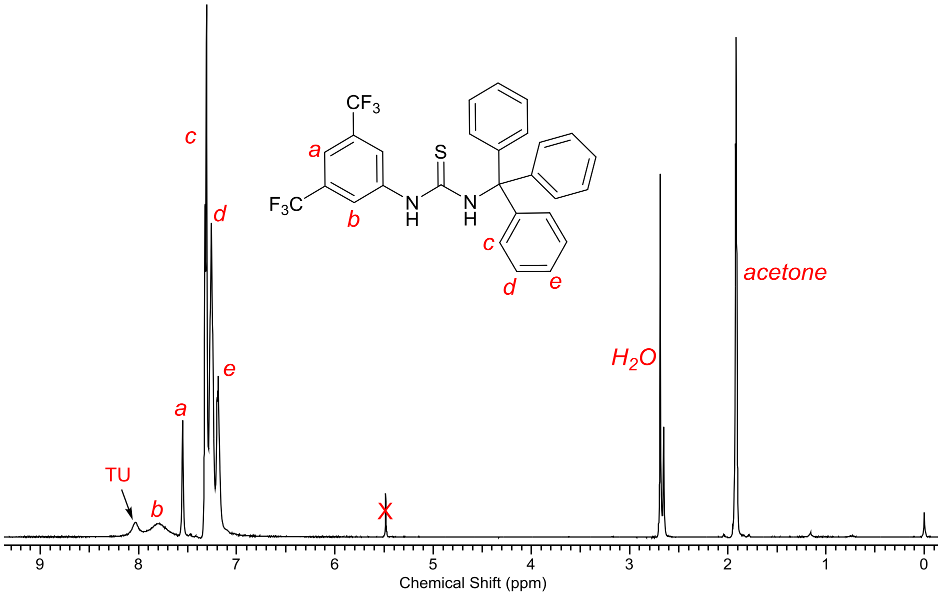

Figure S16. ^{1}H NMR of acyclic catalyst **4** in d_6-acetone.

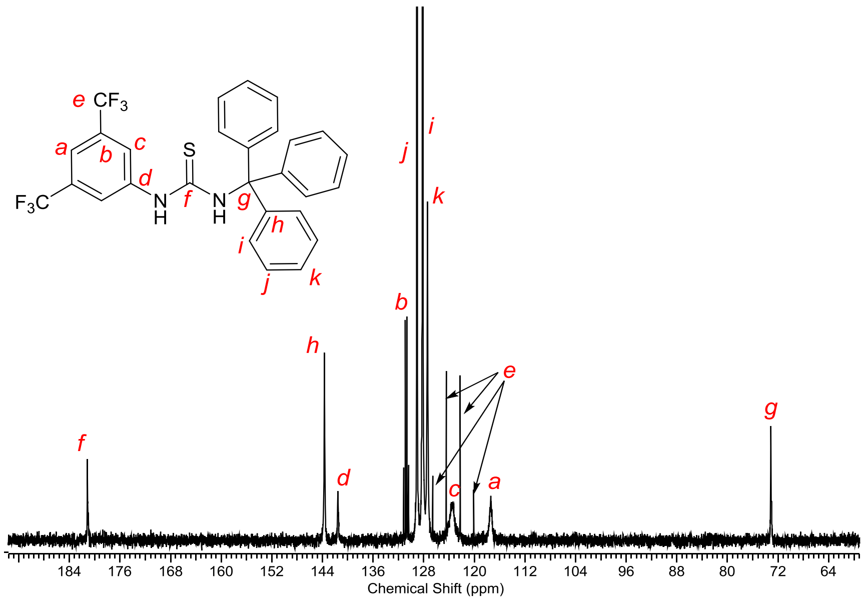

Figure S17. ^{13}C NMR of acyclic catalyst **4** in d_6-acetone.

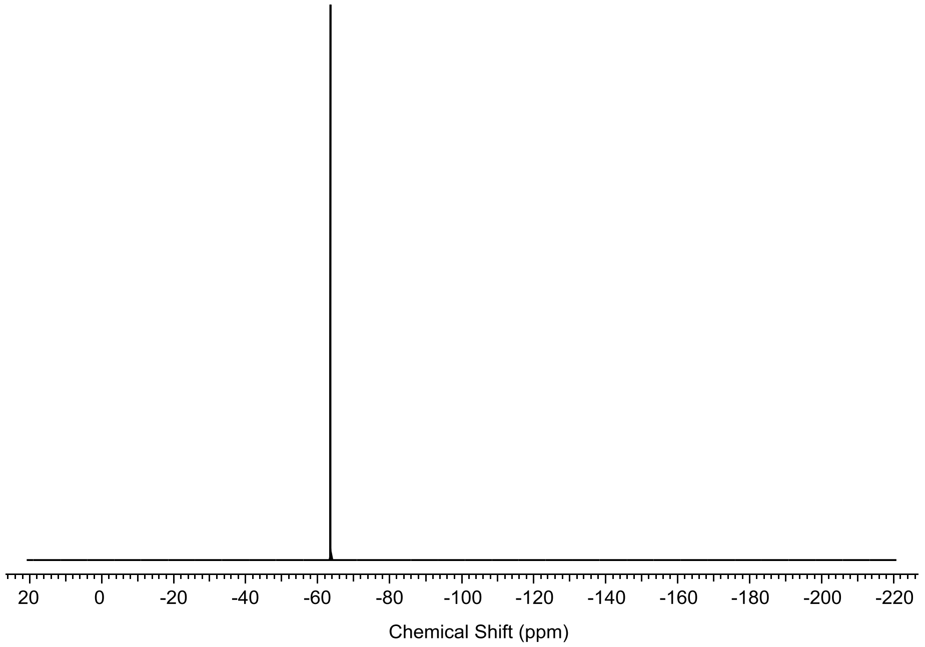

Figure S18. ^{19}F NMR of acyclic catalyst **4** in d_6-acetone.

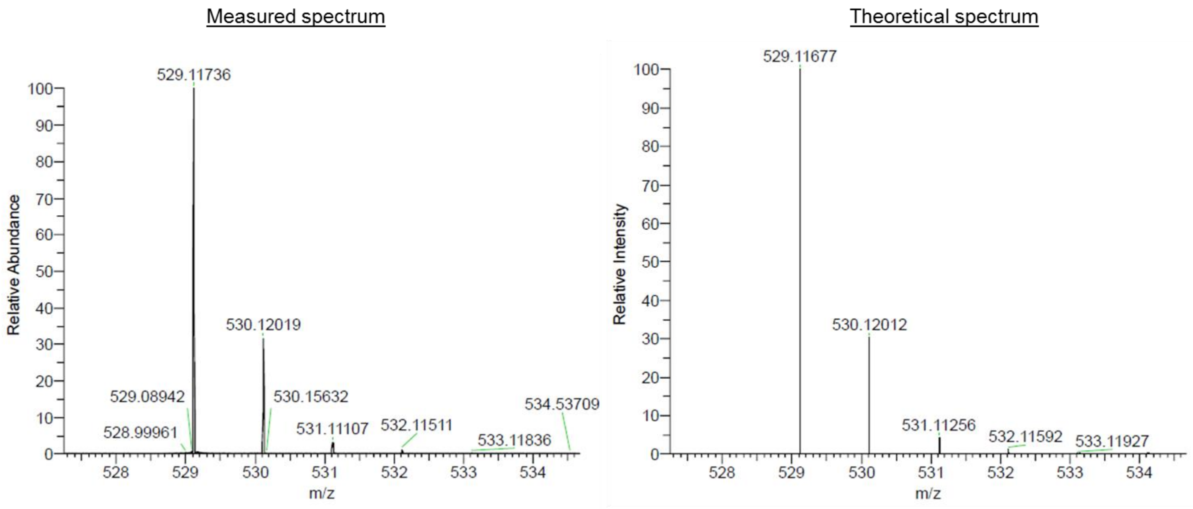

Figure S19. High-resolution ESI-MS characterization (negative mode) of catalyst **4** (left- measured spectrum; right- theoretical spectrum).

Free Thiourea-containing Axle

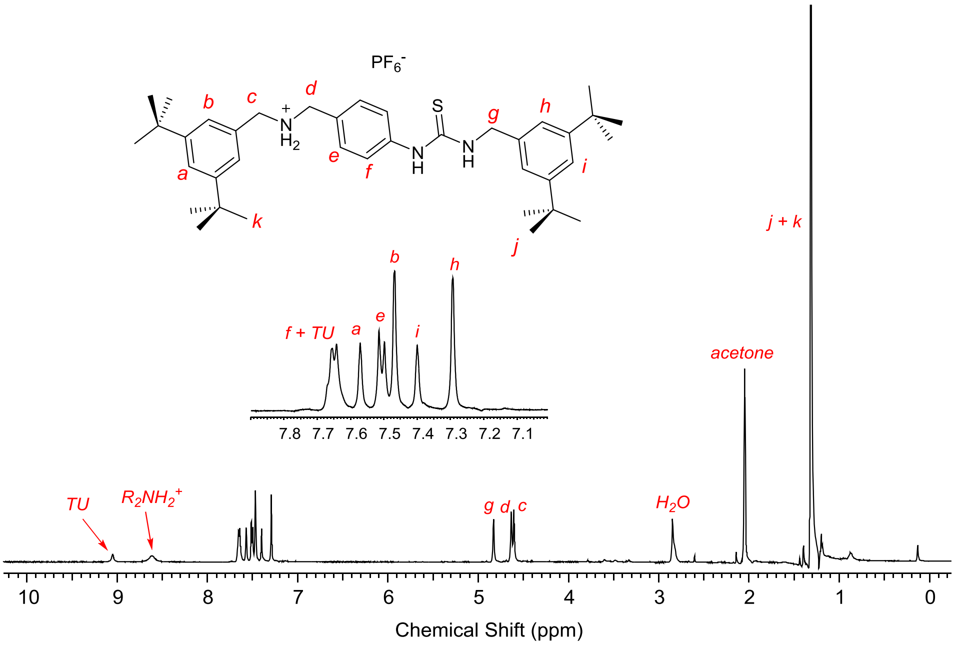

Figure S20. ^{1}H NMR of free thiourea-containing protonated axle in d6-acetone.

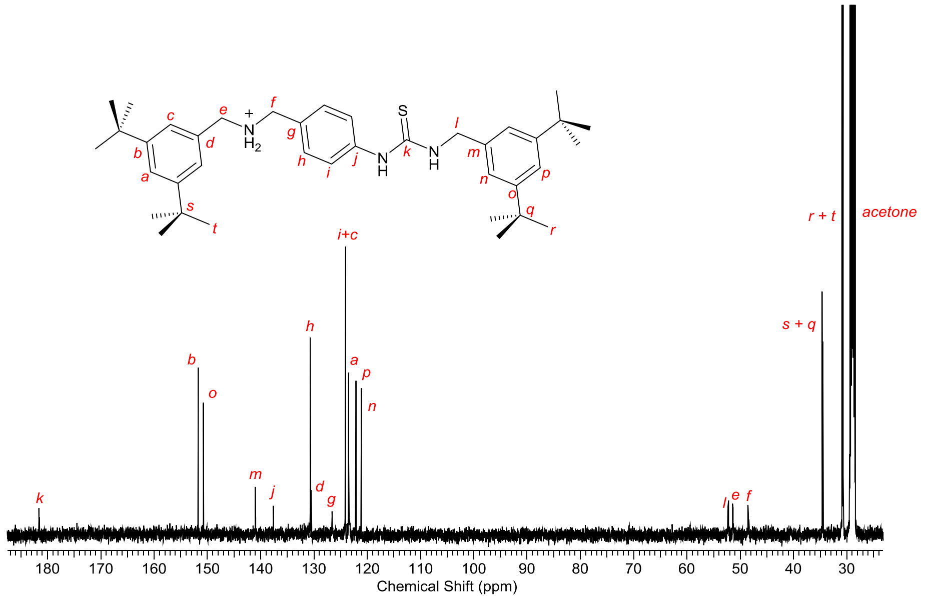

Figure S21. ^{13}C NMR of free thiourea-containing protonated axle in d_6-acetone.

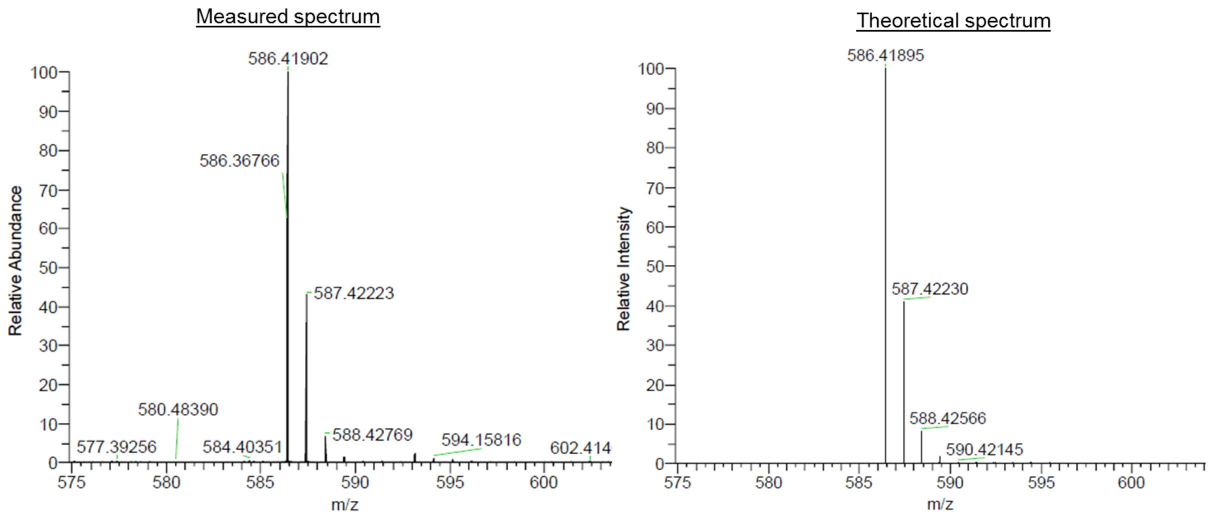

Figure S22. High-resolution ESI-MS characterization (positive mode) of free thiourea-containing protonated axle (left- measured spectrum; right- theoretical spectrum).

Free Triazole-containing Axle

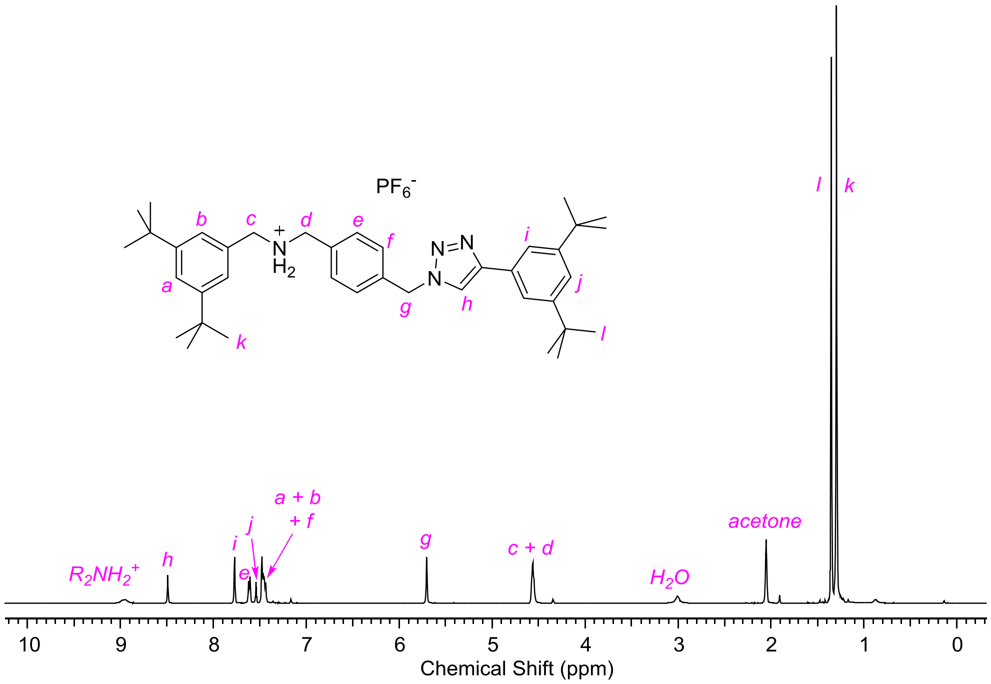

Figure S23. ^{1}H NMR of free thiourea-containing protonated axle in d6-acetone.

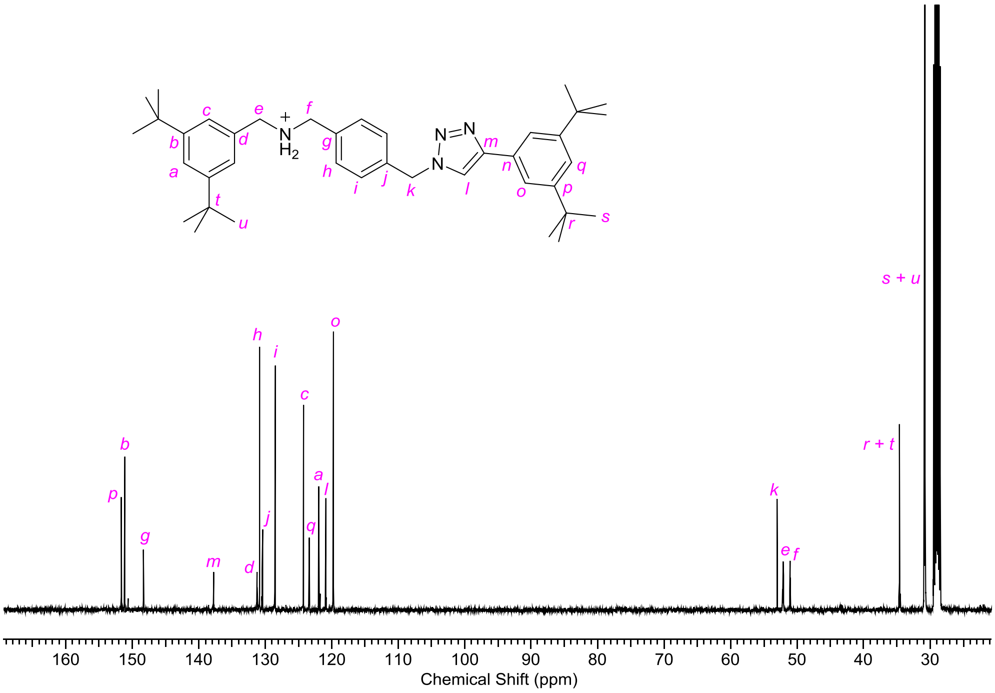

Figure S24. ^{13}C NMR of free thiourea-containing protonated axle in d6-acetone.

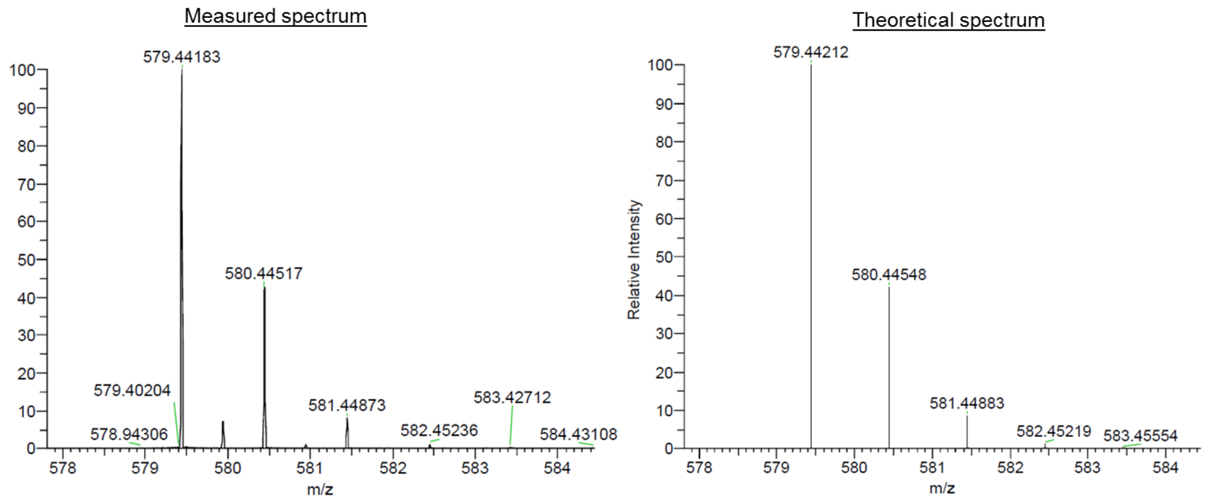

Figure S25. High-resolution ESI-MS characterization (positive mode) of free thiourea-containing protonated axle (left- measured spectrum; right- theoretical spectrum).

S3. Additional Data for Polymerization Experiments

S3.1 Spectra and characterization of PLA

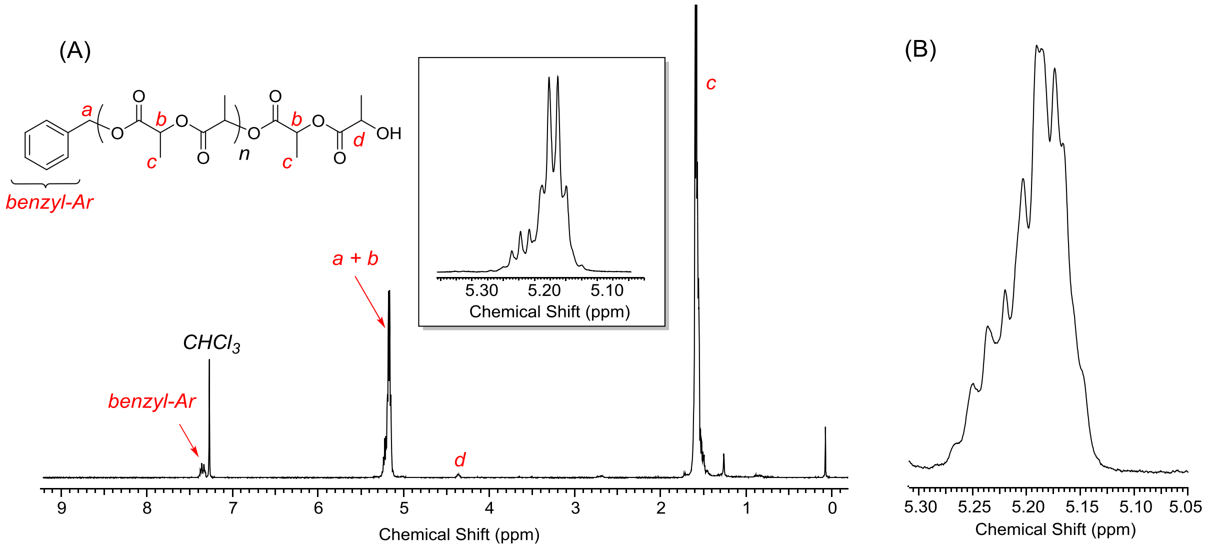

Figure S26. (A) ^{1}H NMR spectrum ($CDCl_3$) of the purified PLA initiated in the presence of rotaxane **1**. Inset: expanded methine region of PLA; (B) Expanded methine region in the ^{1}H NMR spectrum of atactic PLA initiated by $KN(SiMe_3)_2$ and benzyl alcohol in the *absence* of rotaxane **1**.

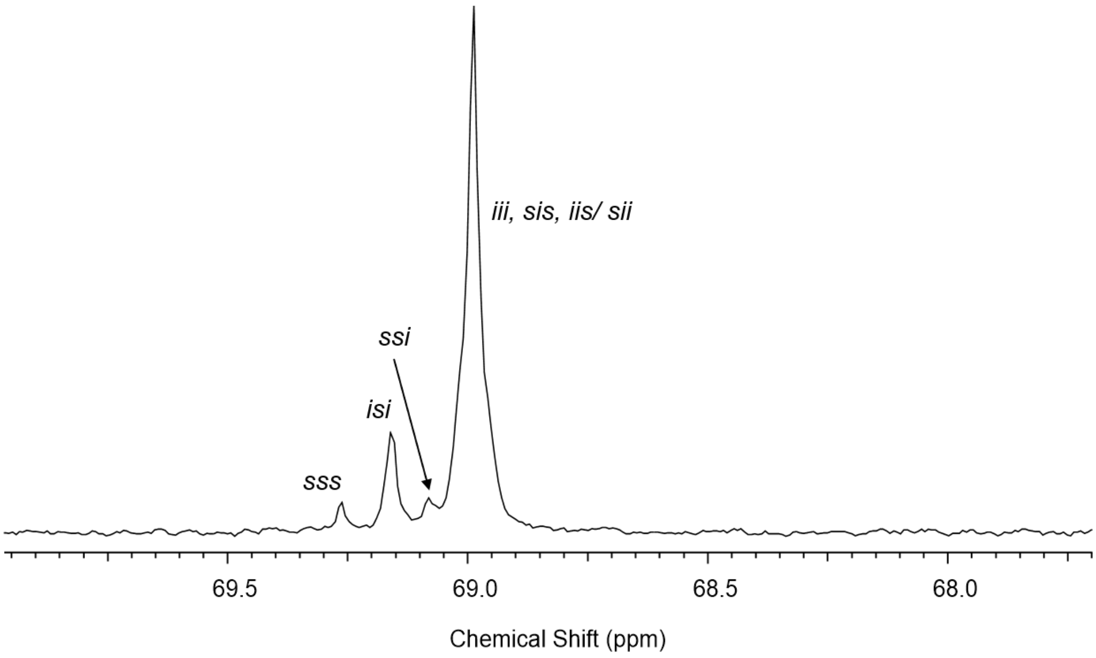

Figure S27. ^{13}C NMR spectrum of the purified isotactic PLA initiated in the presence of rotaxane **1** ($CDCl_3$). Tetrad resonances are labelled. The presence of the small peak at c.a. 69.27 ppm, assigned as the *sss* tetrad resonance, suggests that a small extent of epimerisation occurred during ROP.

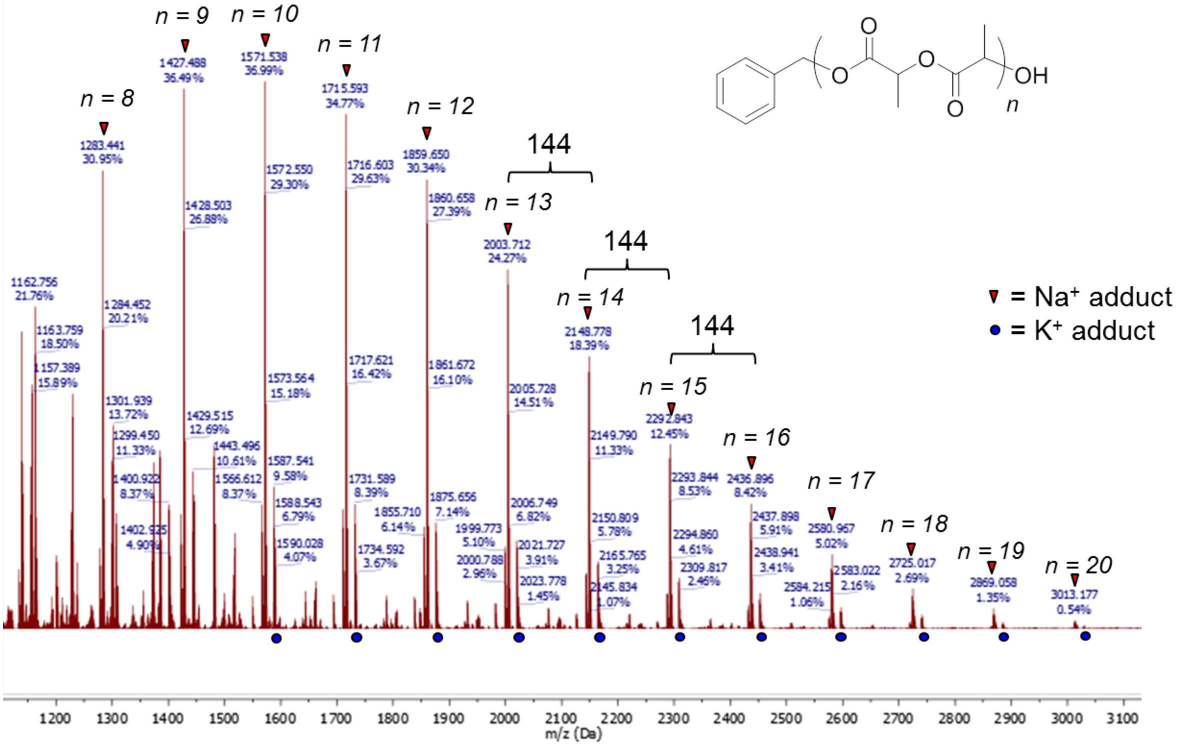

Figure S28. ESI mass spectrum of the crude polymerisation reaction with rotaxane **1** after 7 hours reaction time (25 % monomer conversion from ^{1}H NMR analysis).

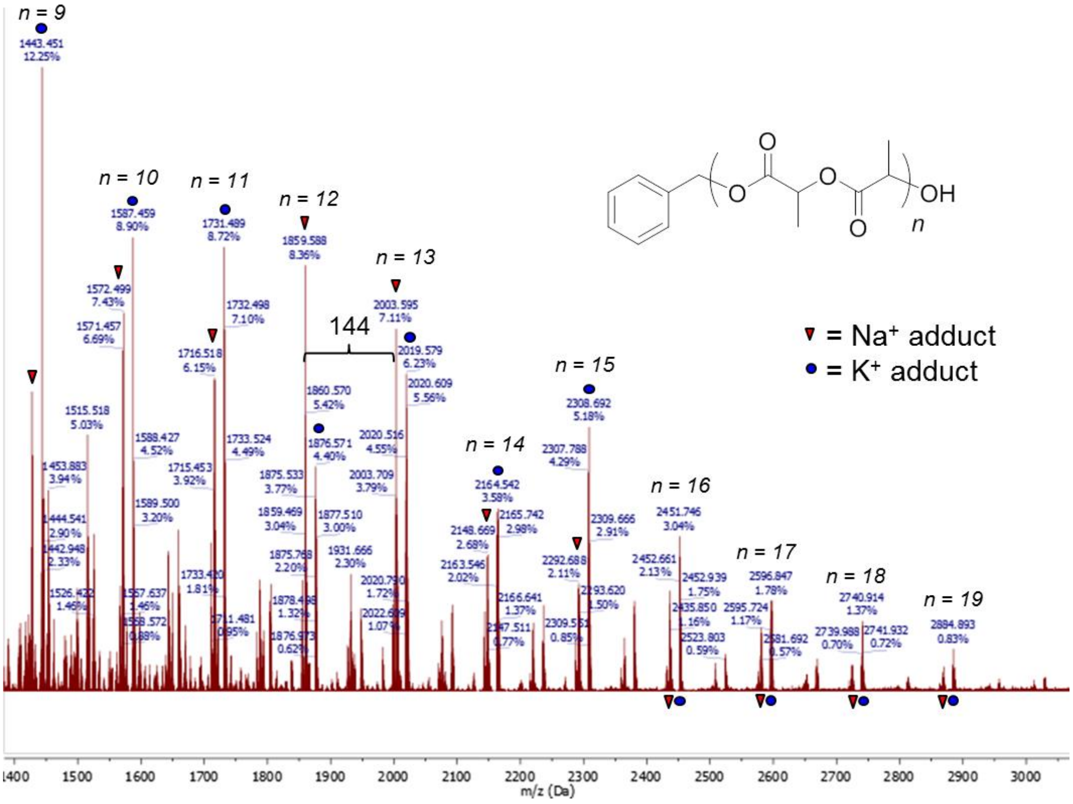

Figure S29. ESI mass spectrum of the crude polymerization reaction with rotaxane **2** after 1 hour reaction time (30 % monomer conversion from ^{1}H NMR analysis).

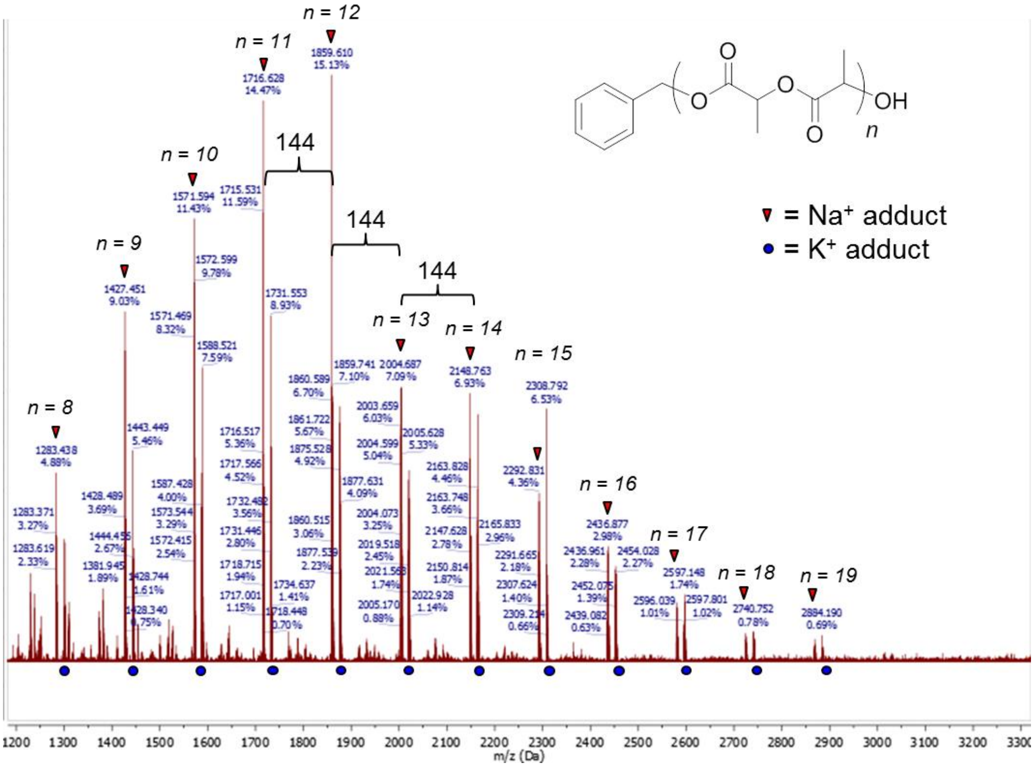

Figure S30. ESI mass spectrum of the crude polymerization reaction with rotaxane **3** after 0.5 hour reaction time (22 % monomer conversion from ^{1}H NMR analysis).

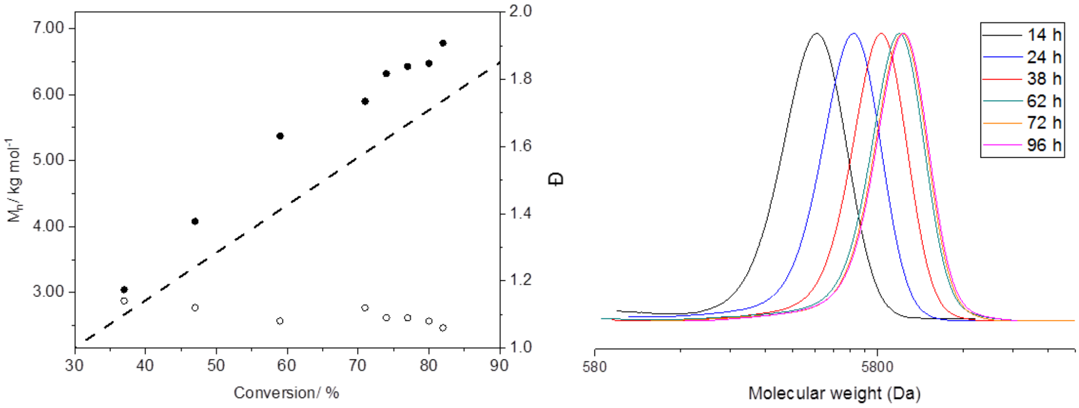

Figure S31. Plots of *M*n (●), theoretical *M*n (-) and *Đ* (○) against percentage conversion (left) and overlay of GPC traces for the polymerization of *rac*-lactide initiated by complex **1** (right).

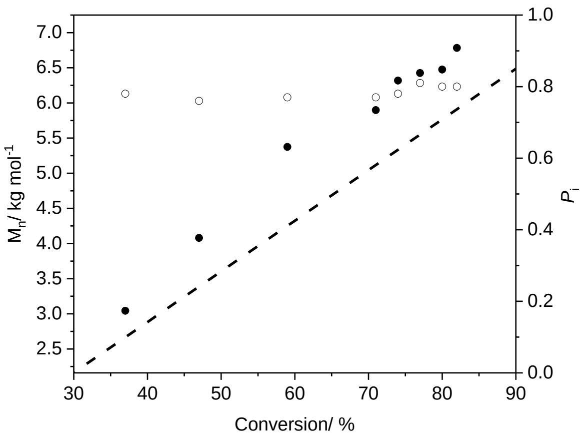

Figure S32. Plots of M_n (●), theoretical M_n (-) and P_i (○) against percentage conversion (left) for polymerization using rotaxane **1** ([lactide] = 1.0 M, T = 298 K, dry THF).

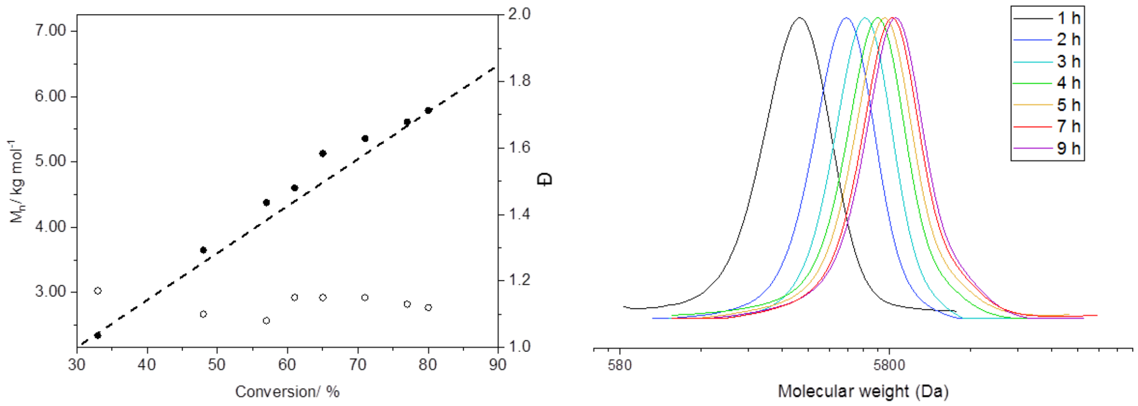

Figure S33. Plots of M_n (●), theoretical M_n (-) and $Đ$ (○) against percentage conversion (left) and overlay of GPC traces for the polymerization of *rac*-lactide initiated by rotaxane **2** (right).

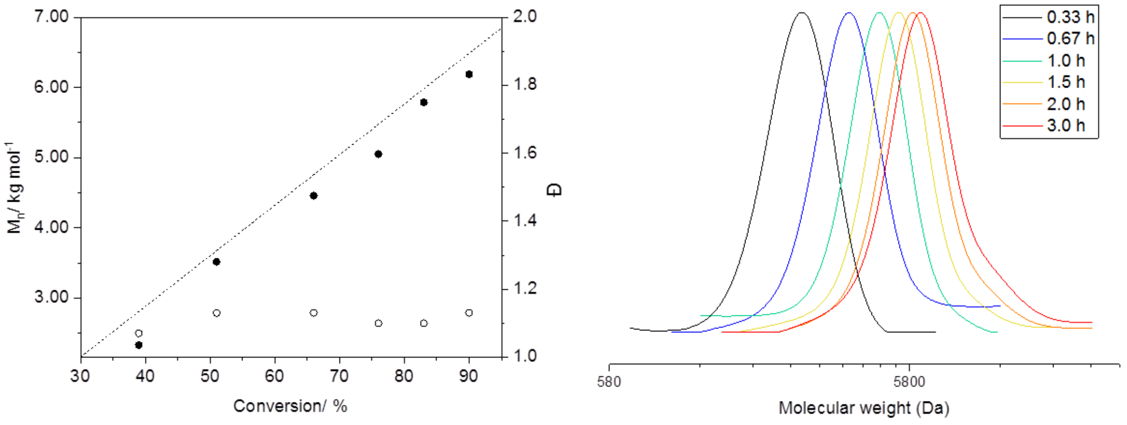

Figure S34. Plots of M_n (●), theoretical M_n (-) and $Đ$ (○) against percentage conversion (left) and overlay of GPC traces for the polymerization of *rac*-lactide initiated by rotaxane **3** (right).

Tetrad	Integration	P_i
sis	0.03	0.75
sii	0.11	0.68
iis	0.07	0.81
iii	0.72	0.80
isi	0.06	0.87
Average (sii, iis, iii, isi)		0.81±0.03

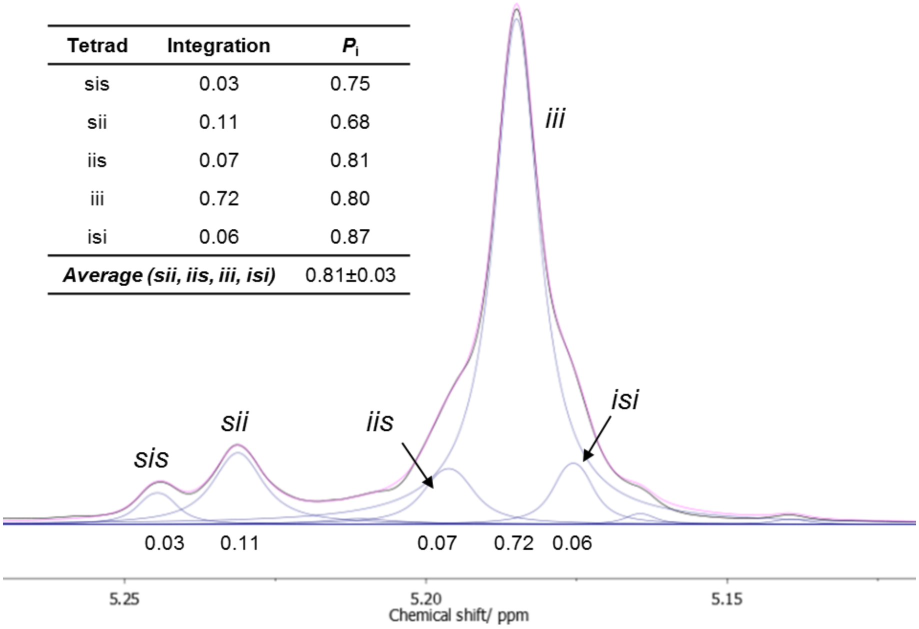

**Stereoerror calculation for CEC mechanism**	**Stereoerror calculation for ESC mechanism**
i i s i i<br>*SS*-*SS*-*SS*-*RR*-*RR*-*RR*	i i s i s<br>*SS*-*SS*-*SS*-*RR*-*SS*-*SS*
Theoretical:<br>sii:isi:iis = 1:1:1	Theoretical:<br>sis:sii:isi:iis = 1:1:2:1

"Rotaxane **1**: sii:isi:iis = 0.11:0.07:0.06 ≈ 2:1:1 suggested CEC is likely predominant"

Figure S35. $^{1}H\{^{1}H\}$ NMR spectrum of the resultant PLA initiated by rotaxane **1** at 298 K (Table 1, entry 1- [LA]/ [rotaxane **1**]/ [KN($SiMe_3$)$_2$]/ [BnOH] = 50:1:1:1), as well as analysis of the defect tetrad resonances for CEC and ESC mechanisms.

Tetrad	Integration	P_i
sis	0.09	0.41
sii	0.17	-
iis	0.17	-
iii	0.25	0.62
isi	0.32	0.67
Average (sis, sii, isi)		0.56 ± 0.10

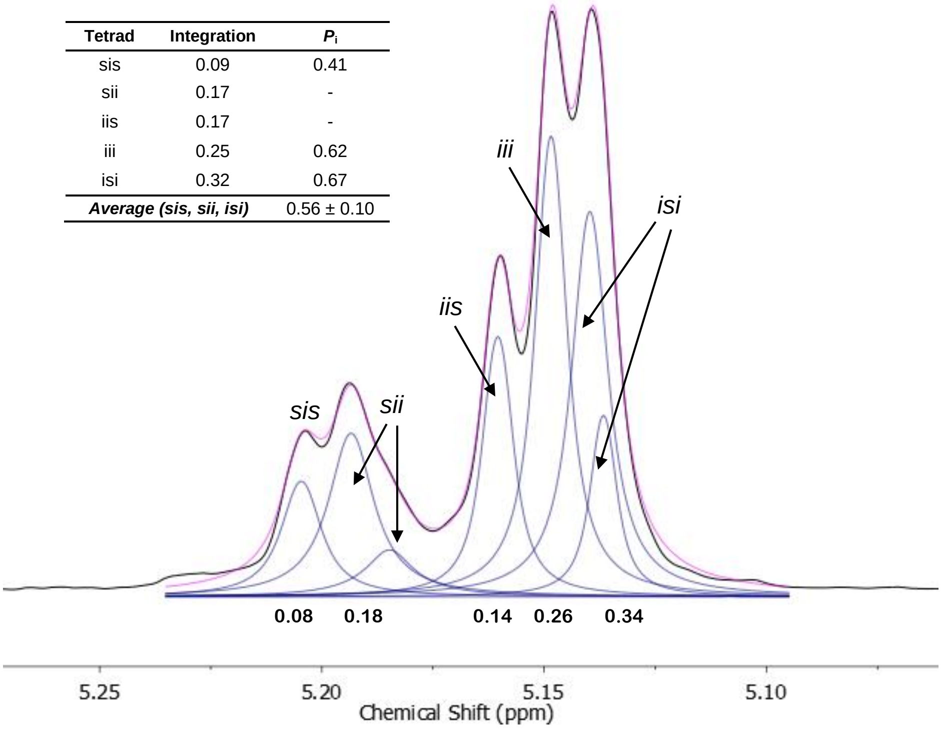

Figure S36. $^1H\{^1H\}$ NMR spectrum of the resultant PLA initiated by in the absence of rotaxane **1** at 298 K (Table 1, entry 2- [LA]/ [rotaxane **1**]/ [$KN(SiMe_3)_2$]/ [BnOH] = 50:0:1:1).

Tetrad	Integration	P_i
sis	0.06	0.64
sii	0.17	-
iis	0.10	0.72
iii	0.39	0.52
isi	0.28	0.45
Average (sii, iii, isi)		0.54 ± 0.06

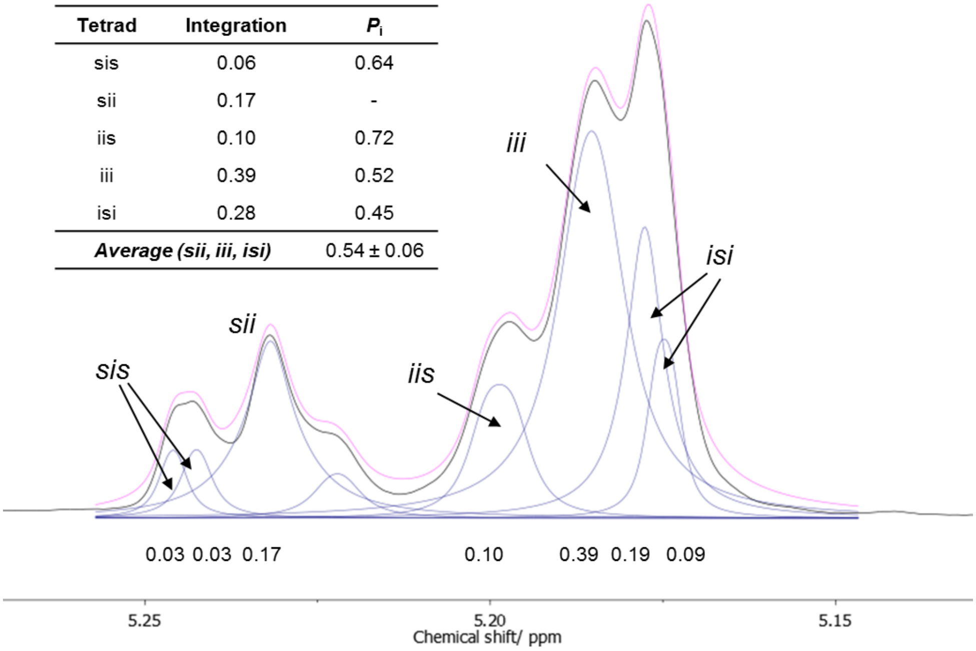

Figure S37. $^1H\{^1H\}$ NMR spectrum of the resultant PLA initiated by in the presence of excess $KN(SiMe_3)_2$ base at 298 K (Table 1, entry 5- [LA]/ [rotaxane **1**]/ [$KN(SiMe_3)_2$]/ [BnOH] = 100:1:2:2).

Tetrad	Integration	P_i
sis	0.04	0.71
sii	0.12	0.60
iis	0.12	0.64
iii	0.57	0.68
isi	0.16	0.69
Average (sis, sii, iis, iii, isi)		0.66 ± 0.02

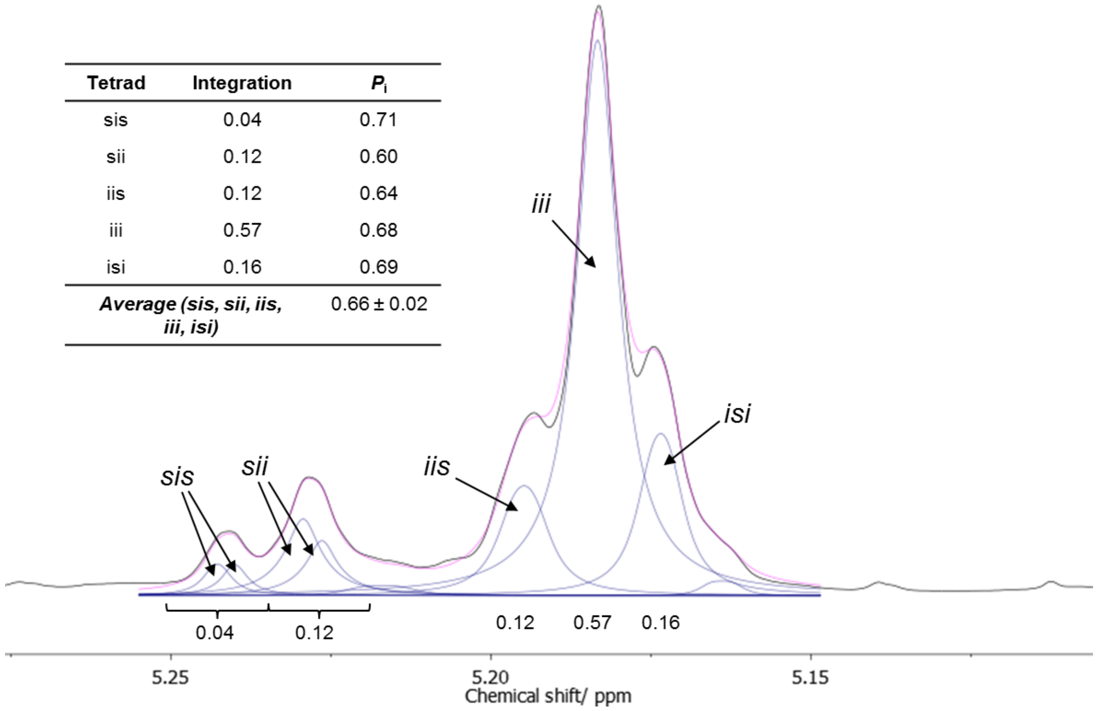

Figure S38. $^{1}H\{^{1}H\}$ NMR spectrum of the resultant PLA initiated by rotaxane **1** at 323 K (Table 1, entry 6- [LA]/ [rotaxane **1**]/ [$KN(SiMe_3)_2$]/ [BnOH] = 50:1:1:1).

Tetrad	Integration	P_i
sis	0.08	0.61
sii	0.14	-
iis	0.06	0.85
iii	0.56	0.67
isi	0.15	0.69
Average (sis, iii, isi)		0.66 ± 0.03

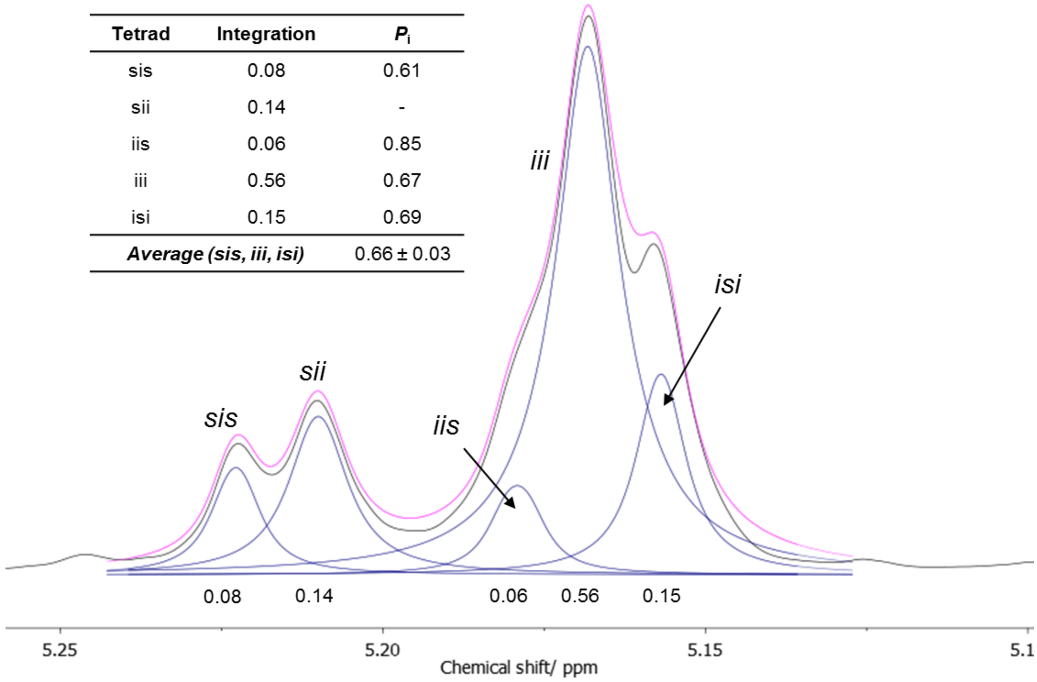

Figure S39. $^{1}H\{^{1}H\}$ NMR spectrum of the resultant PLA initiated by rotaxane **2** at 298 K (Table 1, entry 7- [LA]/ [rotaxane **2**]/ [$KN(SiMe_3)_2$]/ [BnOH] = 50:1:1:1).

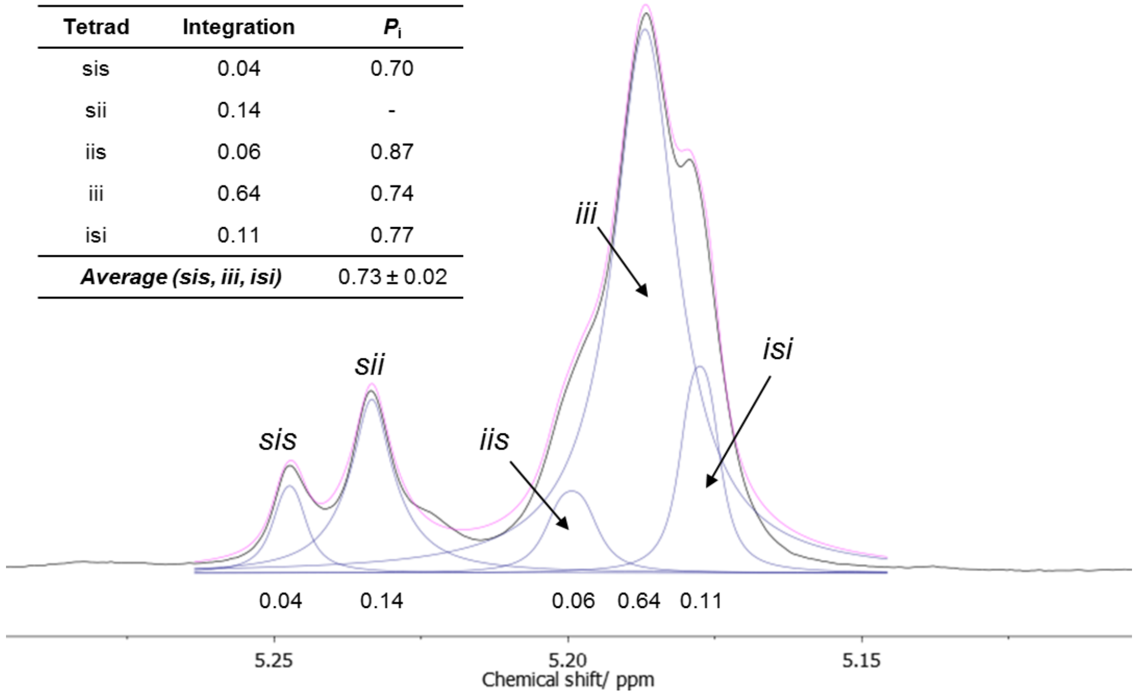

Tetrad	Integration	P_i
sis	0.04	0.70
sii	0.14	-
iis	0.06	0.87
iii	0.64	0.74
isi	0.11	0.77
Average (sis, iii, isi)		0.73 ± 0.02

Figure S40. $^{1}H\{^{1}H\}$ NMR spectrum of the resultant PLA initiated by rotaxane **3** at 298 K (Table 1, entry 8- [LA]/ [rotaxane **3**]/ [KN(SiMe$_3$)$_2$]/ [BnOH] = 50:1:1:1)..

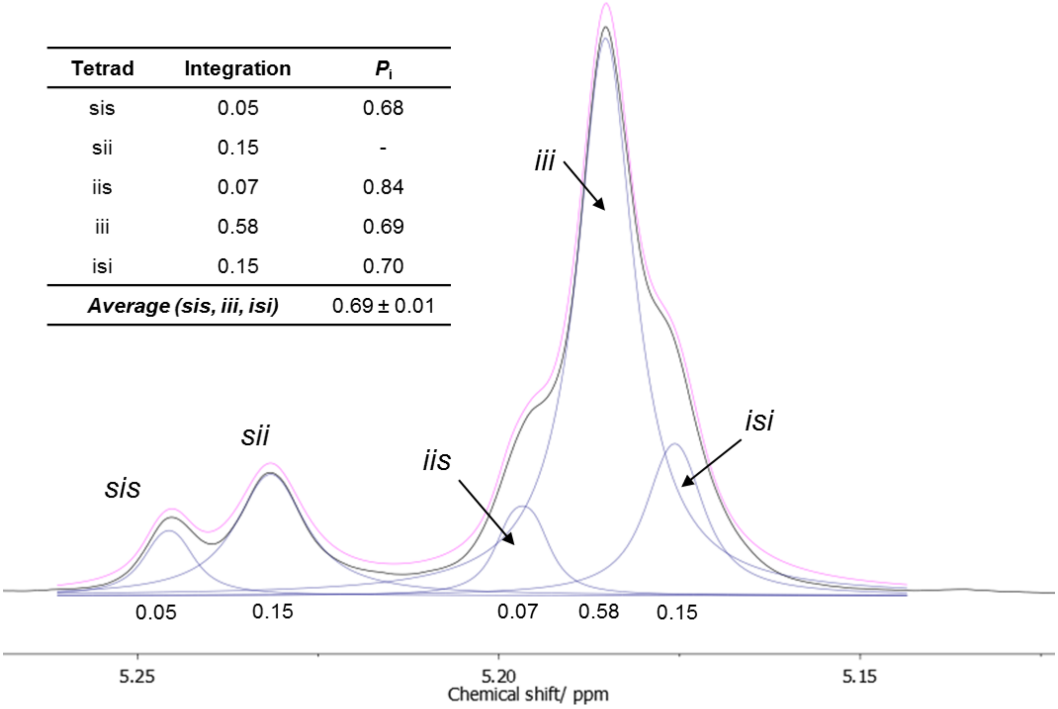

Tetrad	Integration	P_i
sis	0.05	0.68
sii	0.15	-
iis	0.07	0.84
iii	0.58	0.69
isi	0.15	0.70
Average (sis, iii, isi)		0.69 ± 0.01

Figure S41. $^{1}H\{^{1}H\}$ NMR spectrum of the resultant PLA initiated by acyclic catalyst **4** at 298 K (Table 1, entry 6- [LA]/ [catalyst **4**]/ [KN(SiMe$_3$)$_2$]/ [BnOH] = 50:1:1:1).

S3.2 Additional Polymerisation Experiments

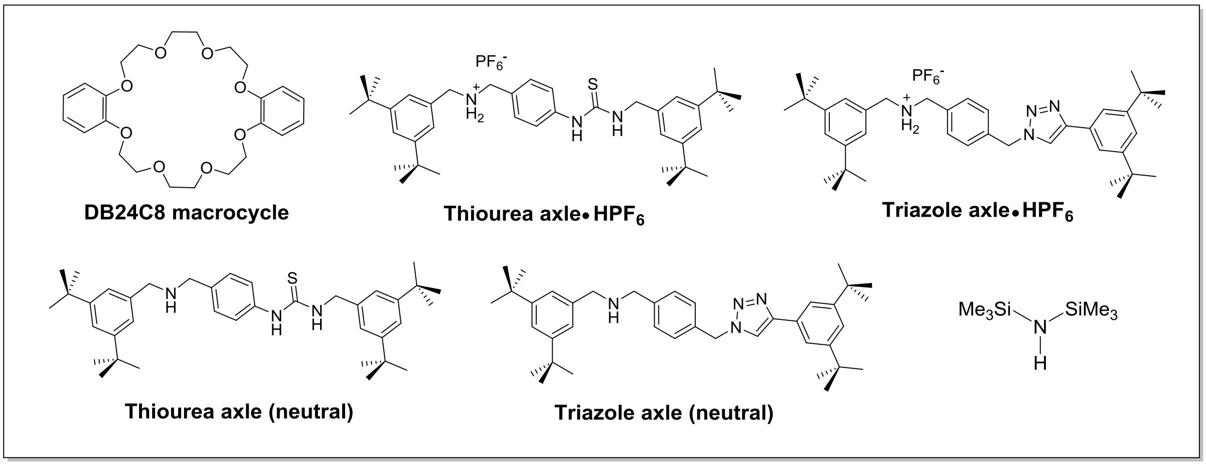

Figure S42. Structures of additional potential catalytic species used.

Table S1. Control Polymerizations of *rac*-Lactide with Potential Catalysts in THF at 298 K.[a]

<table>
<tr><th>S/N</th><th>Catalyst combination</th><th>[LA]/ [cat.]/ [KN(SiMe₃)₂]/ [BnOH]</th><th>t/ h</th><th>conv [b]/ %</th><th>M_n^{GPC} / kDa [c]</th><th>M_n^{calc}/ kDa</th><th>Đ [c]</th><th>P_i [d]</th></tr>
<tr><td>1</td><td>DB24C8 macrocycle</td><td>50: 1: 1: 1</td><td>1</td><td>> 99</td><td>7200</td><td>7200</td><td>1.53</td><td>0.52 ± 0.06</td></tr>
<tr><td>2</td><td>Thiourea axle·HPF6</td><td>50: 1: 1: 1</td><td>48</td><td>0</td><td rowspan="11" colspan="4">Not applicable</td></tr>
<tr><td>3</td><td>Triazole axle·HPF6</td><td>50: 1: 1: 1</td><td>48</td><td>0</td></tr>
<tr><td>4</td><td>Thiourea axle (neutral)</td><td>50: 1: 0: 1</td><td>48</td><td>0</td></tr>
<tr><td>5</td><td>Triazole axle (neutral)</td><td>50: 1: 0: 1</td><td>48</td><td>0</td></tr>
<tr><td>6</td><td>Thiourea axle (neutral) + KPF6</td><td>50: 1: 0: 1</td><td>48</td><td>0</td></tr>
<tr><td>7</td><td>Triazole axle (neutral) + KPF6</td><td>50: 1: 0: 1</td><td>48</td><td>0</td></tr>
<tr><td>8</td><td>HN(SiMe3)2</td><td>50: 1: 0: 0</td><td>48</td><td>0</td></tr>
<tr><td>9</td><td>HN(SiMe3)2</td><td>50: 1: 0: 1</td><td>48</td><td>0</td></tr>
<tr><td>10</td><td>HN(SiMe3)2 + KPF6</td><td>50: 1: 0: 0</td><td>48</td><td>0</td></tr>
<tr><td>11</td><td>HN(SiMe3)2 + KPF6</td><td>50: 1: 0: 1</td><td>48</td><td>0</td></tr>
<tr><td>12</td><td>KPF6</td><td>50: 1: 0: 1</td><td>48</td><td>0</td></tr>
</table>

[a] Performed using [LA] = 1.0 M. [b] Determined by integration of the methine region of the ^{1}H NMR spectrum in $CDCl_3$ (LA, 4.96-5.04 ppm; PLA, 5.10-5.22 ppm); [c] Determined by SEC analysis against polystyrene standards in THF using a Mark-Houwink correction factor of 0.58;[1] [d] Determined by integration of PLA methine tetrads in the deconvoluted ^{1}H{^{1}H} NMR spectrum using values predicted according to Bernoullian statistics.[13] Numbers in parentheses indicate standard errors of the mean P_i values.

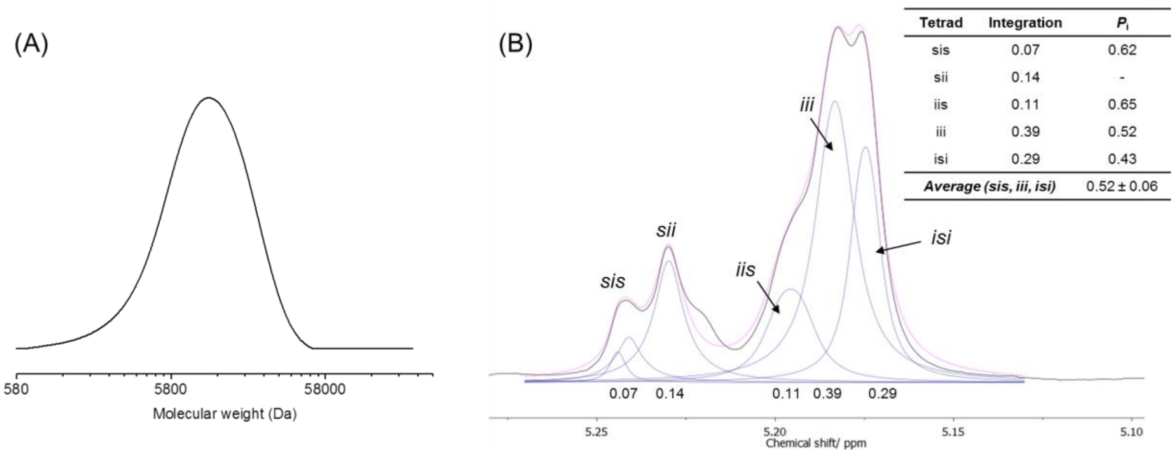

Tetrad	Integration	P_i
sis	0.07	0.62
sii	0.14	-
iis	0.11	0.65
iii	0.39	0.52
isi	0.29	0.43
Average (sis, iii, isi)		0.52 ± 0.06

Figure S43. (A) GPC trace of PLA formed by [LA]/ [**DB24C8** macrocycle]/ [KN(SiMe$_3$)$_2$]/ [BnOH] = 50: 1: 1: 1 (Table S1, entry 1); (B) ^{1}H{^{1}H} NMR spectrum of the resultant PLA initiated by this catalytic combination at 298 K.

S4. Additional NMR Data

S4.1 Additional Spectra

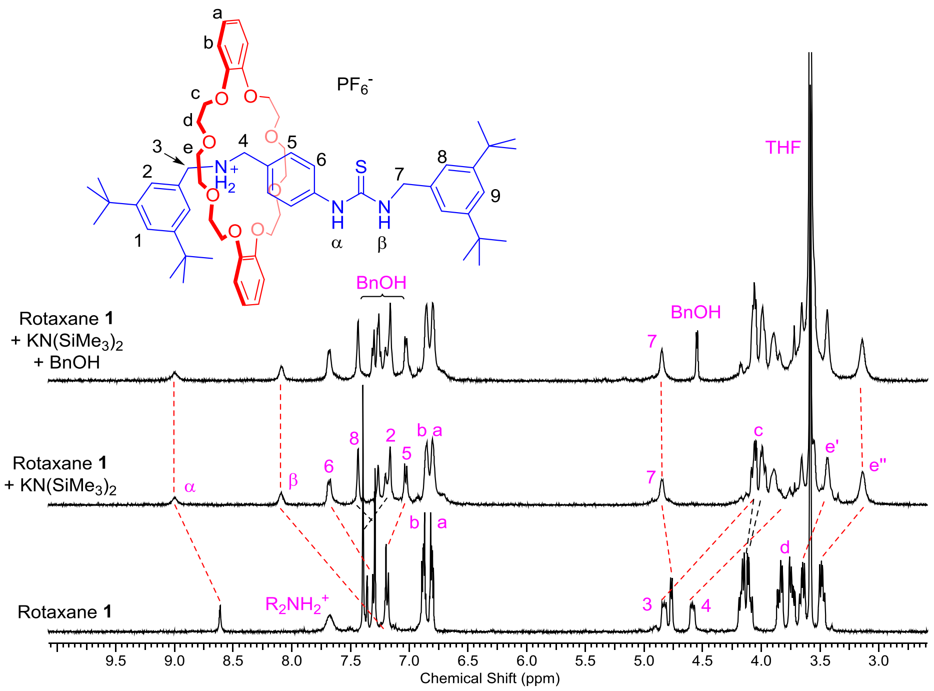

Figure S44. Stacked partial ^{1}H NMR spectra of rotaxane **1** in the presence of 1.0 equivalent of $KN(SiMe_3)_2$ strong base and upon addition of equimolar quantities of BnOH in d_8-THF ([rotaxane **1**] = 2 mM, T = 298 K). Note the spectral broadening, most obviously seen for the signals arising from H_a, H_b and H_7.

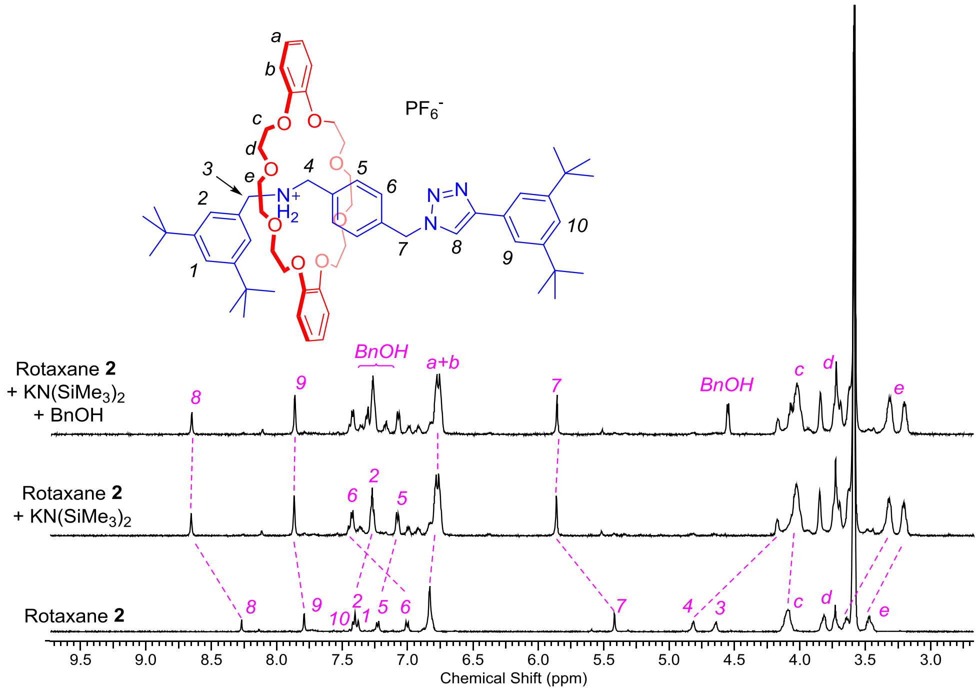

Figure S45. Stacked partial ^{1}H NMR spectra of rotaxane **2** in the presence of 1.0 equivalent of $KN(SiMe_3)_2$ strong base and upon addition of equimolar quantities of BnOH in d_8-THF ([rotaxane **2**] = 2 mM, T = 298 K). Note the retention of the sharp spectra upon addition of base, most clearly seen for peaks arising from H_7, H_8, H_9, H_a and H_b.

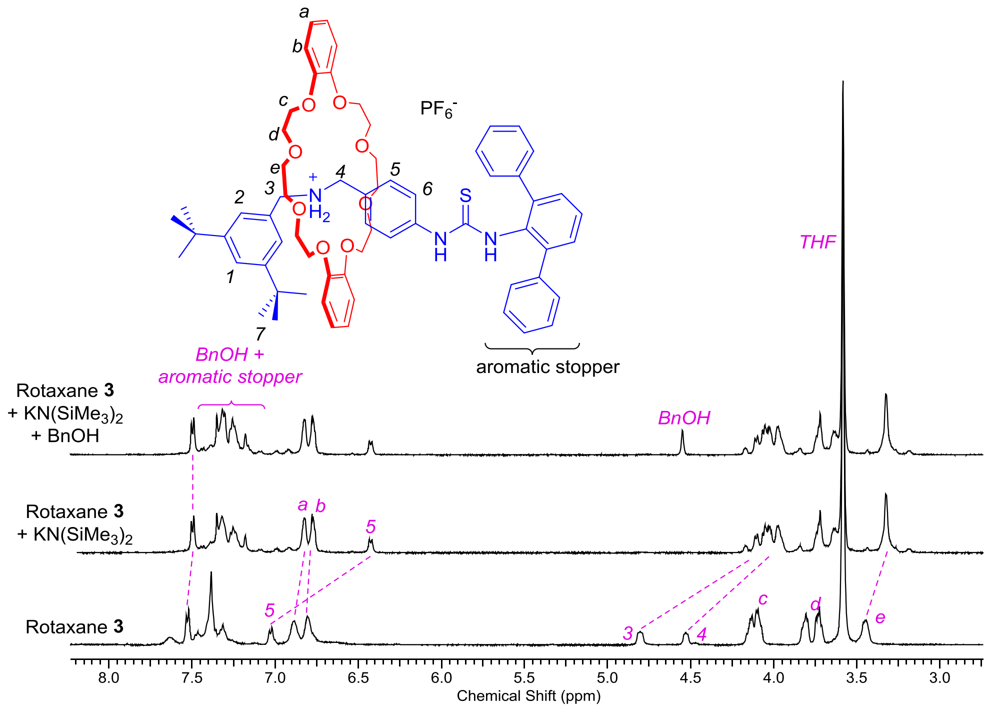

Figure S46. Stacked partial ^{1}H NMR spectra of rotaxane **3** in the presence of 1.0 equivalent of $KN(SiMe_3)_2$ strong base and upon addition of equimolar quantities of BnOH in d_8-THF ([rotaxane **3**] = 2 mM, T = 298 K). The large upfield shifts of H_3 and H_4 are diagnostic that axle ammonium deprotonation occurred upon base addition.

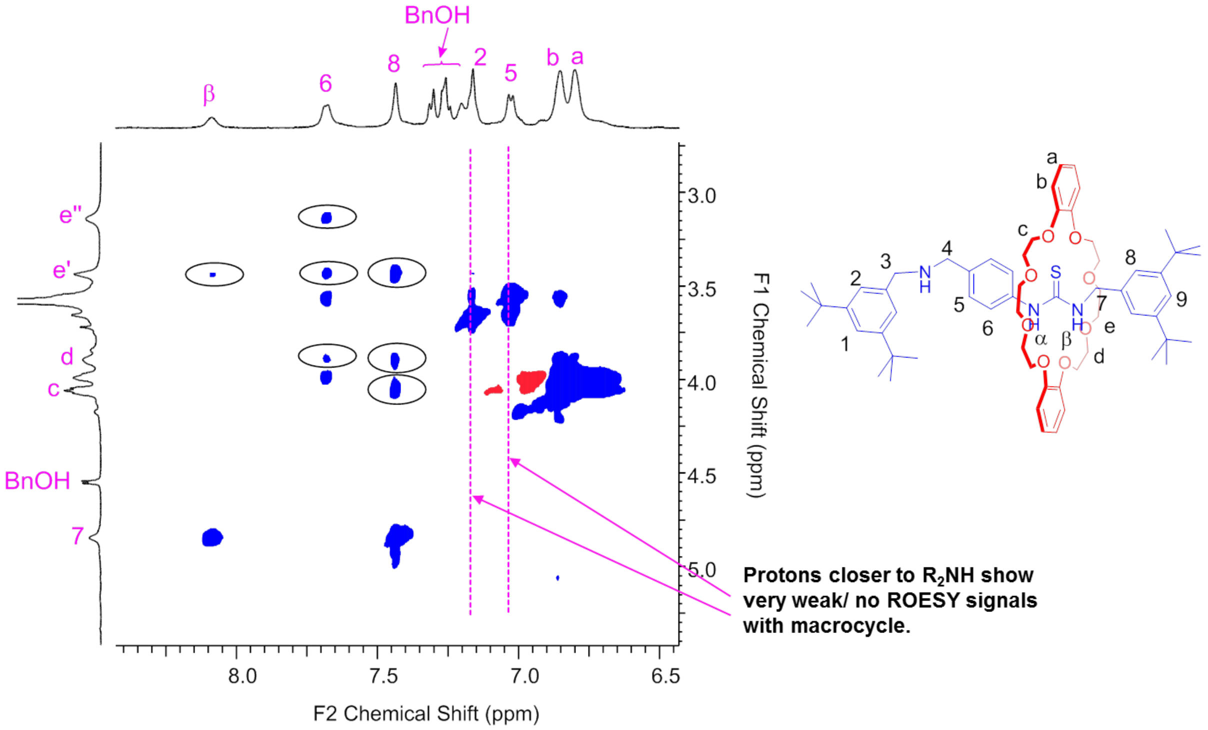

Figure S47. 2D ROESY spectra of the catalytically-active reaction mixture containing rotaxane **1** in d_8-THF. Cross peaks arising from through-space interactions between axle and macrocycle components are circled.

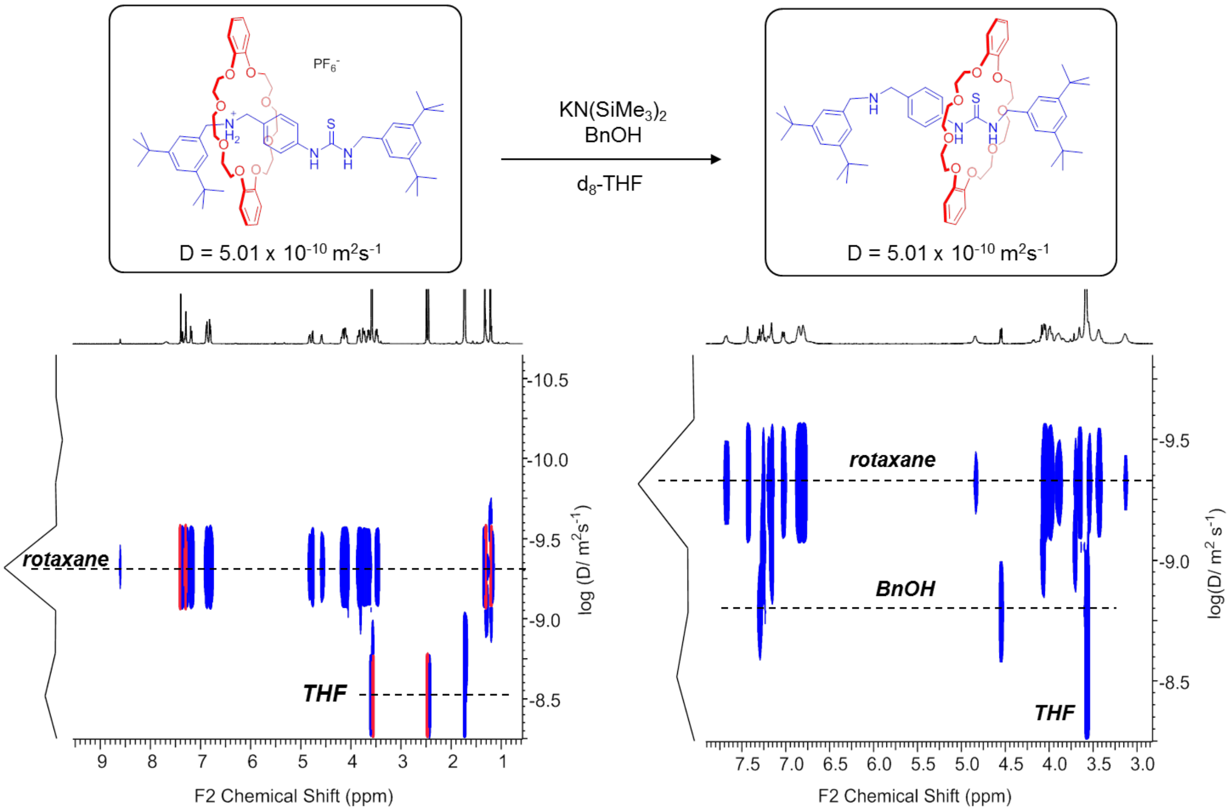

Figure S48. 2D DOSY NMR spectra of protonated rotaxane **1** and its catalytically-active form after addition of $KN(SiMe_3)_2$ and benzyl alcohol.

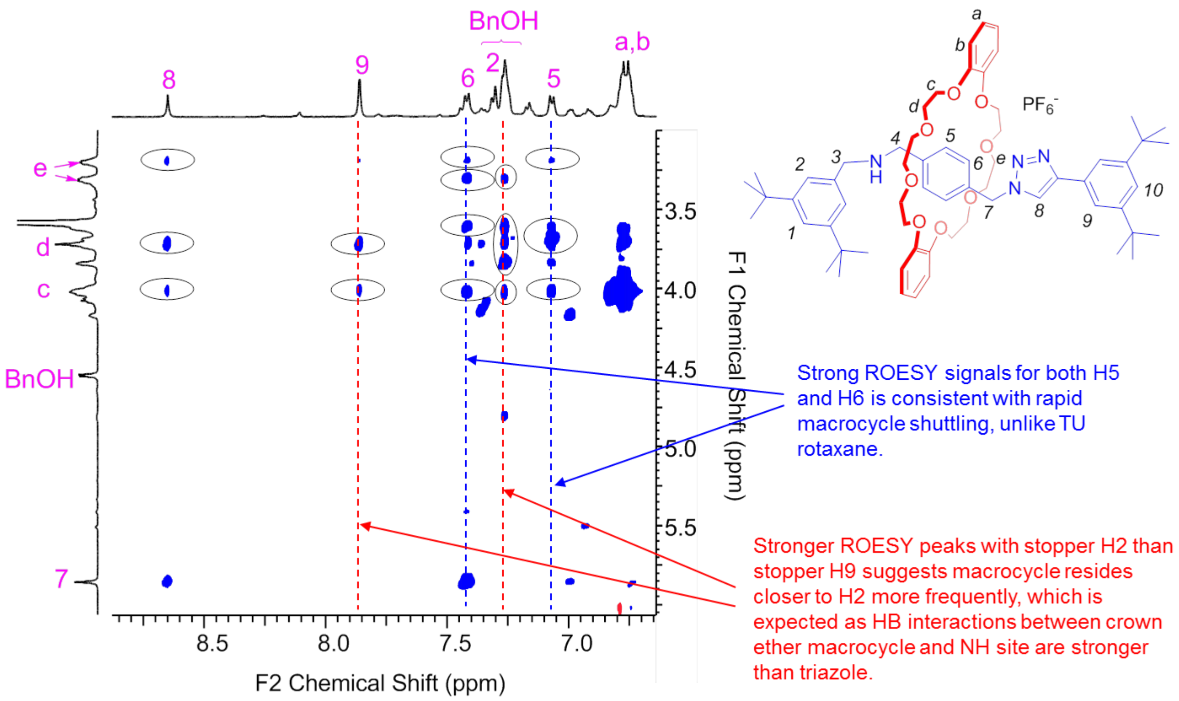

Figure S49. 2D ROESY spectra of the catalytically-active reaction mixture containing rotaxane **2** in d_8-THF. Cross peaks arising from through-space interactions between axle and macrocycle components are circled.

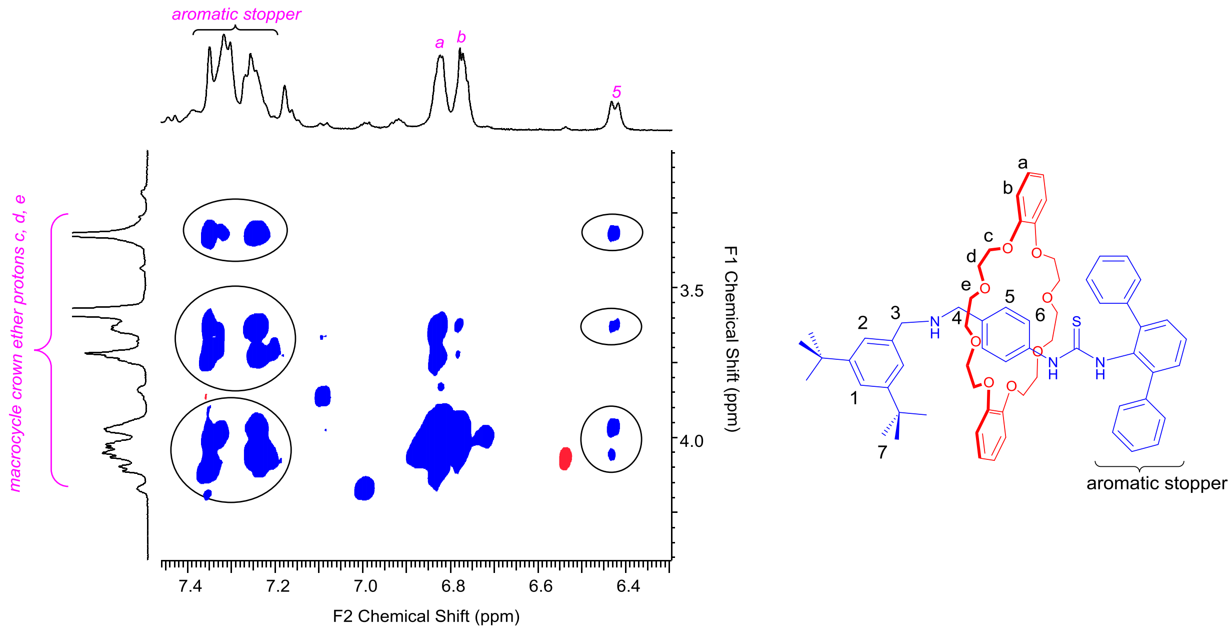

Figure S50. 2D ROESY spectra of the catalytically-active reaction mixture containing rotaxane **3** in d_8-THF. Cross peaks arising from through-space interactions between axle and macrocycle components are circled.

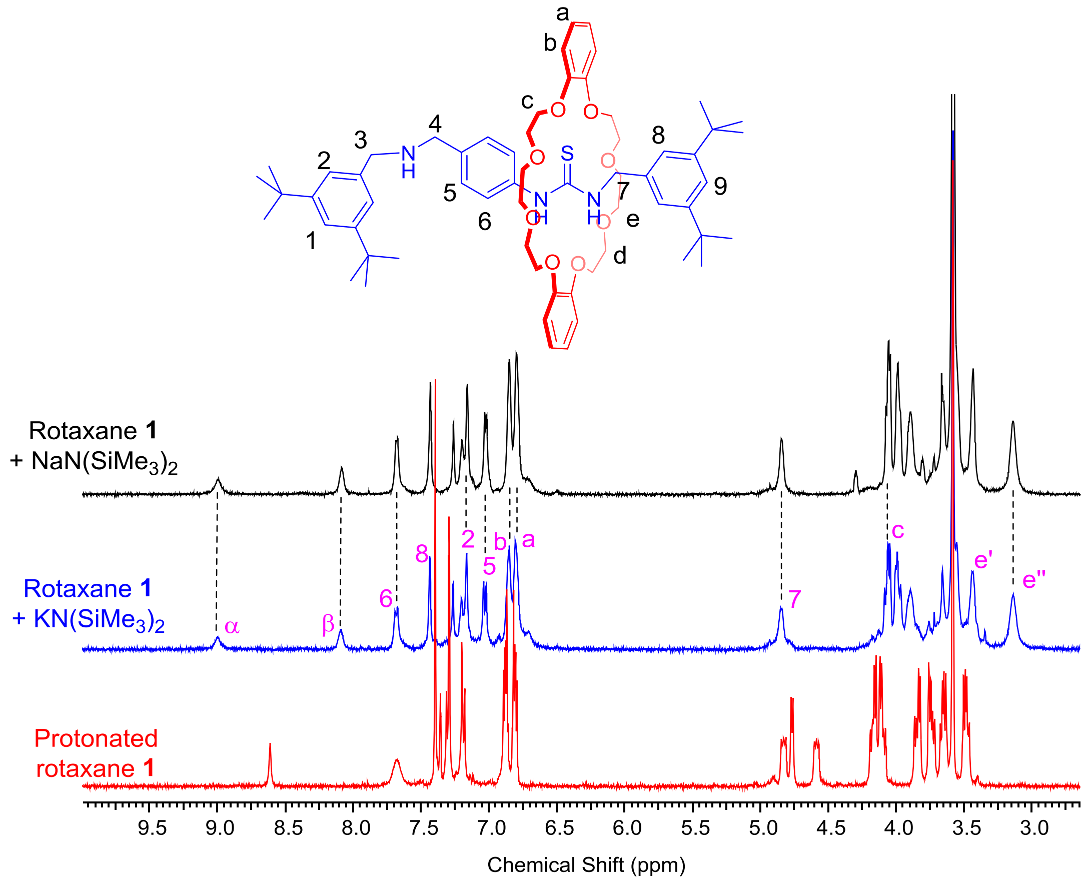

Figure S51. Stacked partial ^{1}H NMR spectra of rotaxane **1** (bottom) in the presence of equimolar quantities of $KN(SiMe_3)_2$ (middle) or its sodium salt $NaN(SiMe_3)_2$ (top) in d_8-THF ([rotaxane **1**] = 2 mM; 298 K).

S4.2 ^{1}H NMR Titration Experiments

^{1}H NMR titration experiments were performed on a Bruker AVIII 500 MHz spectrometer. In a typical experiment, a 1.0 M solution of *rac*-lactide in d_8-THF was added to 0.5 mL of a 2.0 mM solution of the rotaxane at the 298 K in d_8-THF:

For the rotaxane **1** titration, 14 data points corresponding to 0, 1, 2, 3, 4, 5, 6, 8, 10, 15, 20, 25, 30 and 50 equivalents of lactide were obtained. Due to the weaker binding of lactide by rotaxane **2**, a larger excess of lactide was required for plateauing of the binding isotherm to occur. Hence, 15 data points, corresponding to 0, 10, 30, 50, 75, 100, 125, 150, 200, 250, 300, 350, 400, 500 and 600 equivalents of lactide, were obtained.

The binding of lactide with rotaxanes **1** and **2** were found to be fast on the NMR timescale. The values of the observed chemical shift and concentration of lactide were entered into the WinEQNMR2[14] computer programme for every titration point. From initial estimates made of the binding constants and limiting chemical shifts, these parameters were refined using non-linear least-squares analyses to obtain the best fit between empirical and calculated chemical shifts based on a host-guest 1:1 binding stoichiometry (unless otherwise stated), based on equation 6 below. In all cases, convergence of the best fit values of the binding constants and their errors were obtained.

Equation 6:

$$\Delta\delta = \frac{\delta_{HG}}{[H_0]}\left[\frac{1}{2}\left([G_0]+[H_0]+\frac{1}{K_a}\right) - \sqrt{\left([G_0]+[H_0]+\frac{1}{K_a}\right)^2 + 4[G_0][H_0]}\right]$$

where $\Delta\delta$ = observed magnitude of signal shift; δ_{HG} = theoretical maximum chemical shift upon complete saturation of host; $[H_0]$ = concentration of host; $[G_0]$ = concentration of guest; K_a = 1:1 host-guest association constant.

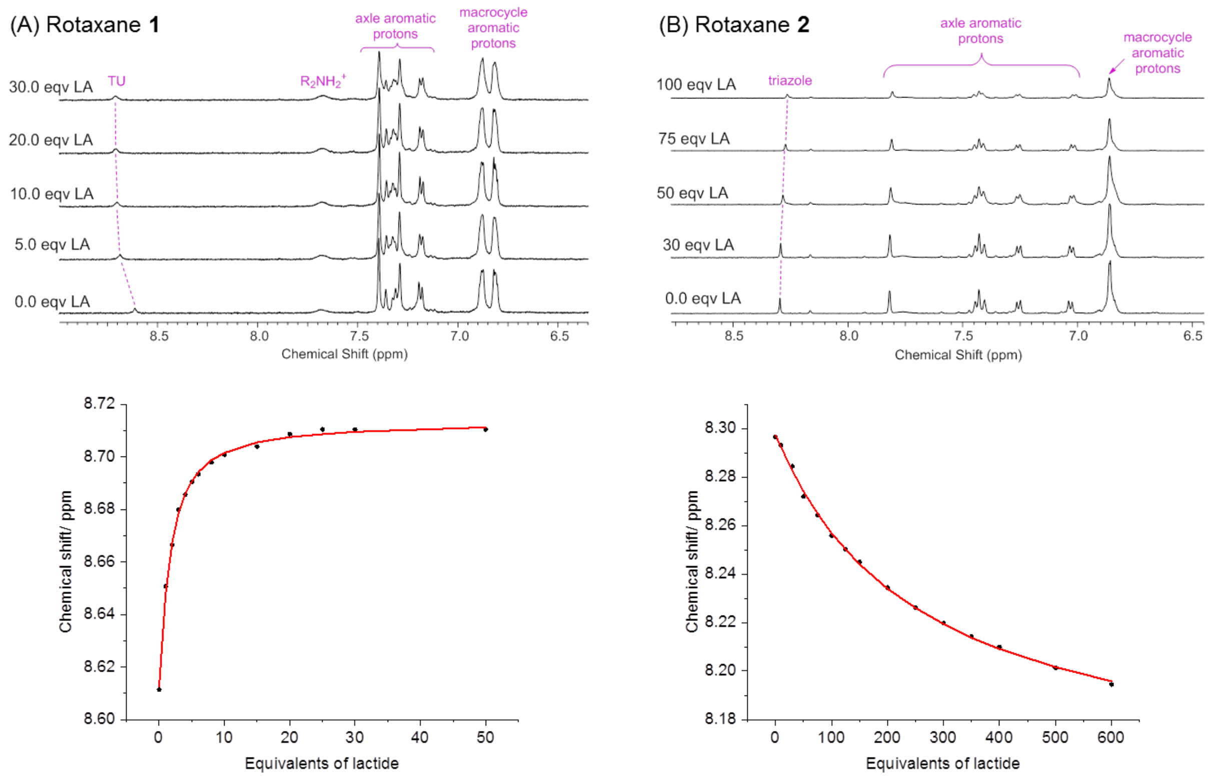

Figure S52. (Top) Stacked partial ^{1}H NMR spectra of protonated (A) rotaxane **1** and (B) rotaxane **2** in the presence of increasing quantities of *rac*-lactide; (Bottom) Binding isotherms obtained by plotting the chemical shifts of a thiourea proton for rotaxane **1** and the triazole proton for rotaxane **2** as a function of increasing quantities of lactide. Empirical data points are represented by the filled dots, while continuous lines represent the calculated binding curves.

S5. References

(1) Baran, J.; Duda, A.; Kowalski, A.; Szymanski, R.; Penczek, S. " Cyclodextrins in Polymer Synthesis: Enantiodiscrimination in Free-Radical Polymerization of Cyclodextrin-Complexed Racemic *N*-Methacryloyl-D,L-phenylalanine Methyl Ester" *Macromol. Rapid. Commun.* **2003**, *18*, 325-330.

(2) Katsumi, I.; Kondo, H.; Yamashita, K.; Hidaka, T.; Hosoe, K.; Yamashita, T.; Watanabe, K. " Studies on Styrene Derivatives I.: Synthesis and Antiinflammatory Activities of α-Benzylidene-γ-butyrolactone Derivatives". *Chem. Pharm. Bull* **1986**, *34*, 121-129.

(3) Tachibana, Y.; Kawasaki, H.; Kihara, N.; Takata, T. "Sequential *O*- and *N*-Acylation Protocol for High-Yield Preparation and Modification of Rotaxanes: Synthesis, Functionalization, Structure, and Intercomponent Interaction of Rotaxanes". *J. Org. Chem.* **2006**, *71*, 5093-5104.

(4) Kim, Y.; Rhee, Y. H.; Park, J. " Redox reaction between benzyl azides and aryl azides: concerted synthesis of aryl nitriles and anilines". *Org. Biomol. Chem.* **2017**, *15*, 1636-1641.

(5) Lipshutz, B. H.; Hageman, M.; Fennewald, J. C.; Linstadt, R.; Slack, E.; Voigtritter, K. " Selective oxidations of activated alcohols in water at room temperature". *Chem. Commun.* **2014**, *50*, 11378-11381.

(6) Corey, E. J.; Fuchs, P. L. " A synthetic method for formyl→ethynyl conversion". *Tet. Lett.* **1972**, *13*, 3769-3772.

(7) Zhang, R.; Hao, X.; Li, X.; Zhou, Z.; Sun, J.; Cao, R. " Soluble Silver Acetylide for the Construction and Structural Conversion of All-Alkynyl-Stabilized High-Nuclearity Homoleptic Silver Clusters". *Cryst. Growth Des.* **2015**, *15*, 2505-2513.

(8) Han, E.-J.; Sun, Y.; Shen, Q.; Chen, Q.-Y.; Guo, Y.; Huang, Y.-G. "Cu-Mediated 2,2,2-trifluoroethylation of terminal alkynes using 1,1-dichloro-2,2,2-trifluoroethane (HCFC-123)". *Org. Chem. Front.* **2015**, *2*, 1379-1387.

(9) Simó Padial, J.; Poater, J.; Nguyen, D. T.; Tinnemans, P.; Bickelhaupt, F. M.; Mecinović, J. " Stabilization of 2,6-Diarylanilinum Cation by Through-Space Cation−π Interactions". *J. Org. Chem.* **2017**, *82*, 9418-9424.

(10) Burns, N. Z.; Witten, M. R.; Jacobsen, E. N. " Dual Catalysis in Enantioselective Oxidopyrylium-Based [5 + 2] Cycloadditions". *J. Am. Chem. Soc.* **2011**, *133*, 14578-14581.

(11) Chadwick, D. J.; Hodgson, S. T. " The protecting–directing role of the trityl group in syntheses of pyrrole derivatives: efficient preparations of 1-*H*-pyrrole-3-carboxylic acid and 3-acyl-, 3-amino-, and 3-bromo-1-tritylpyrroles ". *J. Chem. Soc., Perkin Trans. 1* **1983**, 93-102.

(12) Ferla, S.; Bassetto, M.; Pertusati, F.; Kandil, S.; Westwell, A. D.; Brancale, A.; McGuigan, C. " Rational design and synthesis of novel anti-prostate cancer agents bearing a 3,5-bis-trifluoromethylphenyl moiety". *Bioorg. Med. Chem. Lett.* **2016**, *26*, 3636-3640.

(13) Coudane, J.; Ustariz-Peyret, C.; Schwach, G.; Vert, M. ""More about the stereodependence of DD and LL pair linkages during the ring-opening polymerization of racemic lactide". *J. Polym. Sci. A Polym. Chem.* **2000**, *35*, 1651-1658.

(14) Hynes, M. J. "EQNMR: a computer program for the calculation of stability constants from nuclear magnetic resonance chemical shift data". *J. Chem. Soc., Dalton Trans.* **1993**, 311-312.
